# Foot-based audit of streets adjacent to new light rail stations in Houston, Texas: measurement of health-related characteristics of the built environment for physical activity research

**DOI:** 10.1186/s12889-019-6560-4

**Published:** 2019-02-28

**Authors:** Abiodun O. Oluyomi, Gregory Knell, Casey P. Durand, Clara Mercader, Deborah Salvo, Ipek N. Sener, Kelley Pettee Gabriel, Deanna M. Hoelscher, Harold W. Kohl

**Affiliations:** 1 Environmental Health Service, Section of General Internal Medicine, Department of Medicine, Baylor College of Medicine, One Baylor Plaza, Houston, TX 77030-3411 USA; 2 Department of Health Promotion and Behavioral Science, University of Texas Health Science Center at Houston School of Public Health, Houston, TX USA; 3 Michael & Susan Dell Center for Healthy Living, University of Texas Health Science Center at Houston School of Public Health, Austin, TX USA; 4 Department of Epidemiology, Human Genetics and Environmental Sciences, University of Texas Health Science Center at Houston School of Public Health, Austin, TX USA; 5 Center for Nutrition and Health Research, National Institute of Public Health of Mexico, Cuernavaca, Mexico; 6 Texas A&M Transportation Institute, Texas A&M University System, Austin, TX USA; 7 Department of Women’s Health, Dell Medical School, The University of Texas at Austin, Austin, TX USA; 8 Department of Health Promotion and Behavioral Science, University of Texas Health Science Center at Houston School of Public Health, Austin, TX USA; 9 Department of Kinesiology & Health Education, College of Education, University of Texas at Austin, Austin, TX USA

**Keywords:** Active commute, Physical activity, Environmental audit, Built environment, Urban health

## Abstract

**Background:**

Active travel to and from a transit station may provide significant amounts of physical activity and improve health. The ease with which people can traverse the distance to the transit station may impede or support active travel. Therefore, transit stations that have features that are supportive of utilitarian physical activity would be desirable. This study aimed to characterize the built environment surrounding new light rail transit (LRT) stations in the City of Houston, Texas.

**Methods:**

In 2014, we used a series of systematic protocols and a standardized environmental audit instrument, the Analytic Audit Tool, to collect data on segments (streets) that surround 22 LRT stations that were being newly built. Using Geographic Information System (GIS), we assembled all the segments that intersect a 0.25-mile circular buffer around each station for the audit exercise. Several 3- to 4-member teams of trained auditors completed the audit exercise on a subset of these identified segments. Our analysis were descriptive in nature. We provided the frequency distributions of audited features across the study area. We also followed an original algorithm to produce several composite index scores for our study area. The composite index score is indicative of the prevalence of physical activity friendly/unfriendly features in the study area.

**Results:**

In all, we audited a total of 590 segments covering a total of 218 US Census blocks, and eight City of Houston super neighborhoods. Findings suggest the environment around the new LRT stations may not be supportive of physical activity. In general, the audited segments lacked land use integration; had abandoned buildings, had uneven sidewalks; were not bike-friendly, had minimal presence of public-recreational facilities that would support physical activity; and had significant physical disorder. Notably, certain attractive and comfort features were frequently to usually available.

**Conclusions:**

Current findings, which will be compared to follow-up audit data, can be useful for future researchers and practitioners interested in the built environment around LRT stations.

**Electronic supplementary material:**

The online version of this article (10.1186/s12889-019-6560-4) contains supplementary material, which is available to authorized users.

## Background

In recent years there has been considerable interest in the role of mass (public) transit use (e.g. bus, subway, light rail, etc.) as a source of utilitarian physical activity [1–5]. The assumption is that transit use typically requires some additional travel to and from a transit stop or station (first mile/last mile phenomenon). If this additional travel is physically active (walking or biking), then potentially significant amounts of physical activity can be accrued by transit riders to improve one’s health [1–5].

Intuitively, transit use involving active travel to and from stops or stations requires an environment surrounding the stop or station that is supportive of utilitarian physical activity. For instance, if the immediate area surrounding a transit stop lacks sidewalks or bike lanes, it would be difficult for potential users to access the stop/station by walking or biking [6]. Despite scientific progress in the field of active travel and the built environment, there is a lack of empirical work that seeks to specifically characterize the micro built environment attributes immediately surrounding newly implemented transit stops or stations using environmental audits. The lack of detailed data regarding mass transit station environments is a major limitation toward understanding the potential for transit use as a contributor to active travel and physical activity at large [7]. All transit users must interact with the environment surrounding the transit stop, as it is the only common environment that a diverse user base with unique origins and destinations can all be guaranteed to come into contact with. Therefore, it is important to understand the area adjacent to transit stations and its potential influence on transit use and the travel mode to and from it.

Environmental audits are used to determine the availability of built environment features that may promote or hinder the ability of walkers and/or cyclists to reach and depart transit stops [8–12]. These are on-the-ground assessments of the characteristics of the built environment conducted by trained auditors using standardized protocols and validated instruments. As opposed to macro-scale techniques, such as the use of secondary geographic datasets (e.g. road centerline and parcel data), audits require in-person observation to provide a rich and detailed micro-scale characterization of features of the environment [13]. These features include, for example, sidewalk and bike lane availability, street crossings, building condition and street lights, etc.

The goal of this study was to adapt an existing foot-based audit method for characterizing the built environment surrounding 22 new light rail stations in Houston, Texas, in terms of the features known to be suitable for public transit use, active travel (walking or biking), and physical activity at large. This work was conducted as part of the Houston Travel-Related Activity in Neighborhoods (TRAIN) Study [14]. Briefly, the TRAIN Study was a longitudinal natural experiment that assessed the overall impact of light rail transit expansion on physical activity behaviors. This methodological work is important as it will allow us to test, in future inferential analysis of TRAIN Study data, whether baseline built environment characteristics differentially influence (i.e., moderate) the effects of the light rail transit expansion with transit use and active travel and physical activity behaviors. This descriptive work will also set the stage to draw comparisons between baseline and 4-year follow up audit measures, to assess any significant changes over time in the micro-built environment of the surroundings of these new stations, to answer questions such as whether adding new transit infrastructure detonates local built environmental changes over time (ripple effect). Our specific objective for the current analysis was, therefore, to establish a baseline micro-scale built environment profile of the new Houston light rail stations’ surroundings.

## Methods

A systematic protocol, with detailed standard operating procedures, was developed to conduct the environmental audit, which is detailed below. The protocol described in this report was completed as part of the Houston TRAIN Study baseline data collection activities.

### Train study methods

The TRAIN Study, taking place in the fourth largest city in the United States, Houston, Texas, was a longitudinal natural experiment designed to assess the impact of large-scale public transportation improvements on physical activity behaviors. The TRAIN Study rationale and setting have been described elsewhere [14]. Briefly, the Metropolitan Transit Authority of Harris County (METRO), the agency overseeing public transit in Harris County, which includes the City of Houston, opened three new light rail transit (LRT) lines in December 2013 and May 2015, resulting in 15 miles of new LRT lines and 22 new stations. The existing LRT line which opened in 2004 was a 7.5-mile 16-station line. The three new lines are North, East, and Southeast lines. These new lines run through primarily residential and light commercial areas, and the population served is primarily minority race/ethnicity (Black/African American and Hispanic), and low income; the same population subgroups which are more likely to be physically inactive [15, 16], overweight or obese [17], and suffer from chronic health conditions [18–22].

### Environmental audit protocols

All environmental audit activities occurred during the 2014 spring and summer months. Although the three new lines were originally scheduled to open in 2014, the North line opened slightly ahead of schedule, in December 2013, while East and Southeast lines opened in May 2015. To successfully complete the audit exercise, the following four tasks were defined: 1) conduct auditor training and setting up for field work, 2) establish the audit locations, 3) define audit block and segments, and 4) collect, enter, and clean the audit data. These tasks are described in detail below.

### Auditor training

We recruited seven public health graduate students, who had previous fieldwork coursework and experience, to perform the environmental audit. We used the Analytic Audit Tool for the audit (http://activelivingresearch.org/analytic-audit-tool-and-checklist-audit-tool. Accessed: 2019-01-23. [Archived by WebCite® at http://www.webcitation.org/75enysuWH]). The Analytic Audit Tool (AA-Tool) is used to understand the relations between street-scale environments and physical activity. The AA-Tool has been validated for use in conducting environmental audits in other settings (e.g., residential neighborhoods) [23], and already successfully used in previous studies [24, 25]. The AA-Tool covers five major domains, collected at the micro-environment level: land use, transportation, facilities, signage, and social environment. Auditors were trained on the use of the AA-Tool using a customized training manual that was developed by the TRAIN study team. Auditor training occurred on two consecutive days. On the first day, we ran an in-house four-hour training session, facilitated by the co-investigators, where we explained the objectives of the parent study and discussed all the topics in the AA-Tool. On the second day, the training session was completed in the field at two pre-selected blocks that were located near a light rail transit station. In order to establish conformity of audit assessment, we allowed different teams to audit the same segment separately, and thereafter compared notes in real time during the training field visit. However, we did not compute any inter-rater reliability statistics. At the conclusion of the training, based on observations by the co-investigators during the training session, specific auditors were selected to lead 3- to 4-member teams. Each team audited one segment at a time, and between two to three teams went to the field together on any given data collection day. The teams were identified at the beginning of each audit session, with one lead auditor for each team.

### Audit locations

Following auditor training and prior to data collection, the audit locations were pre-selected. We used geographic information systems (GIS) analysis in ArcGIS 10.1 (Esri, Redlands, CA) to create a 0.25-mile circular buffer around each of the new light rail transit stations (*n* = 22) along the newly developed 15-mile long light rail lines. We selected 0.25 miles in order to provide a sufficient sample of street segments available to audit, while maintaining feasibility and practicality for the auditors on the ground. A 0.25-mile buffer is approximately a 5-min walk and therefore a manageable distance for the auditors (considering time and physical constraints) between selected segments within the buffer.

### Identifying relevant geographies – Blocks, segment, super neighborhoods

**Fig. 1:**
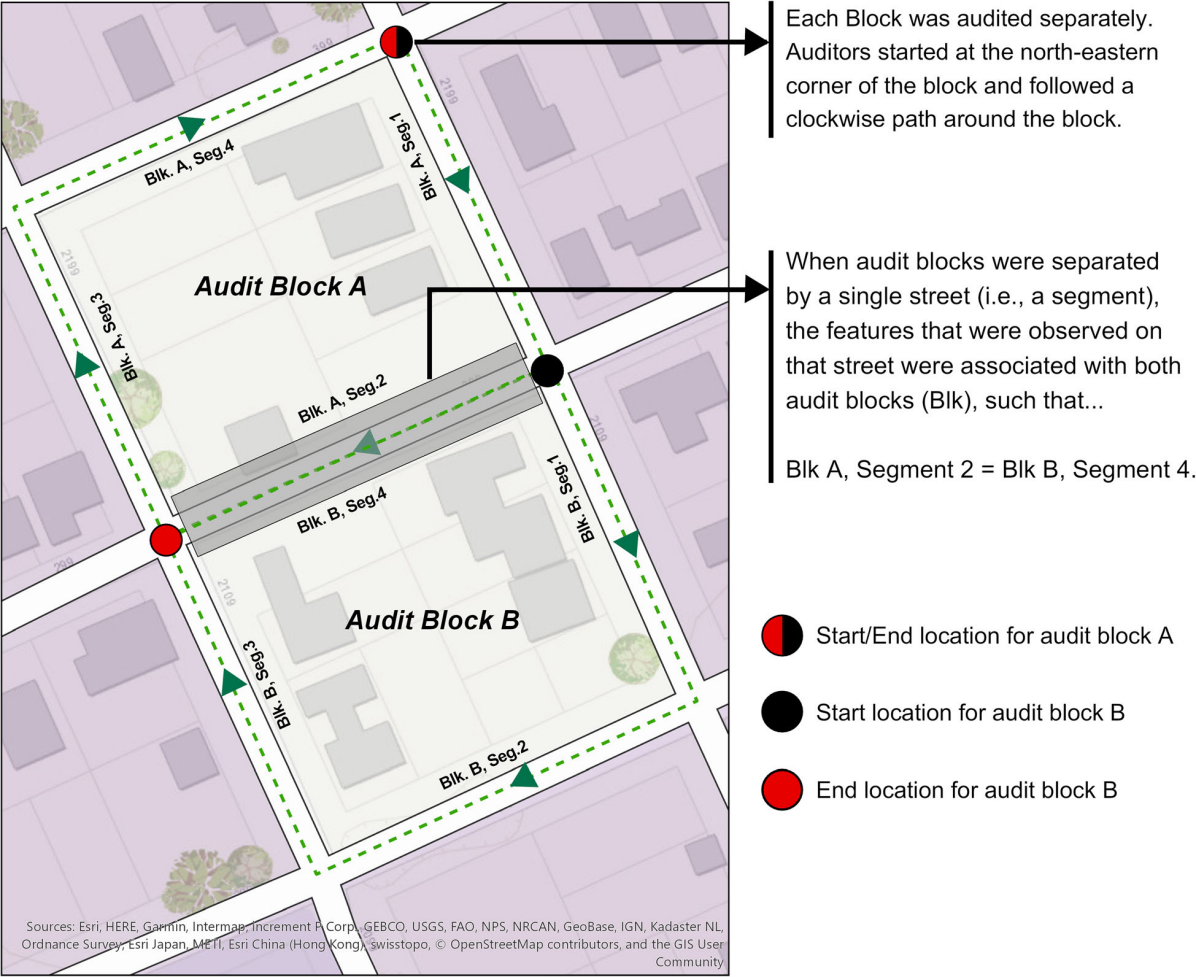
Representation of a typical audit experience when auditing adjacent blocks

**Fig. 2:**
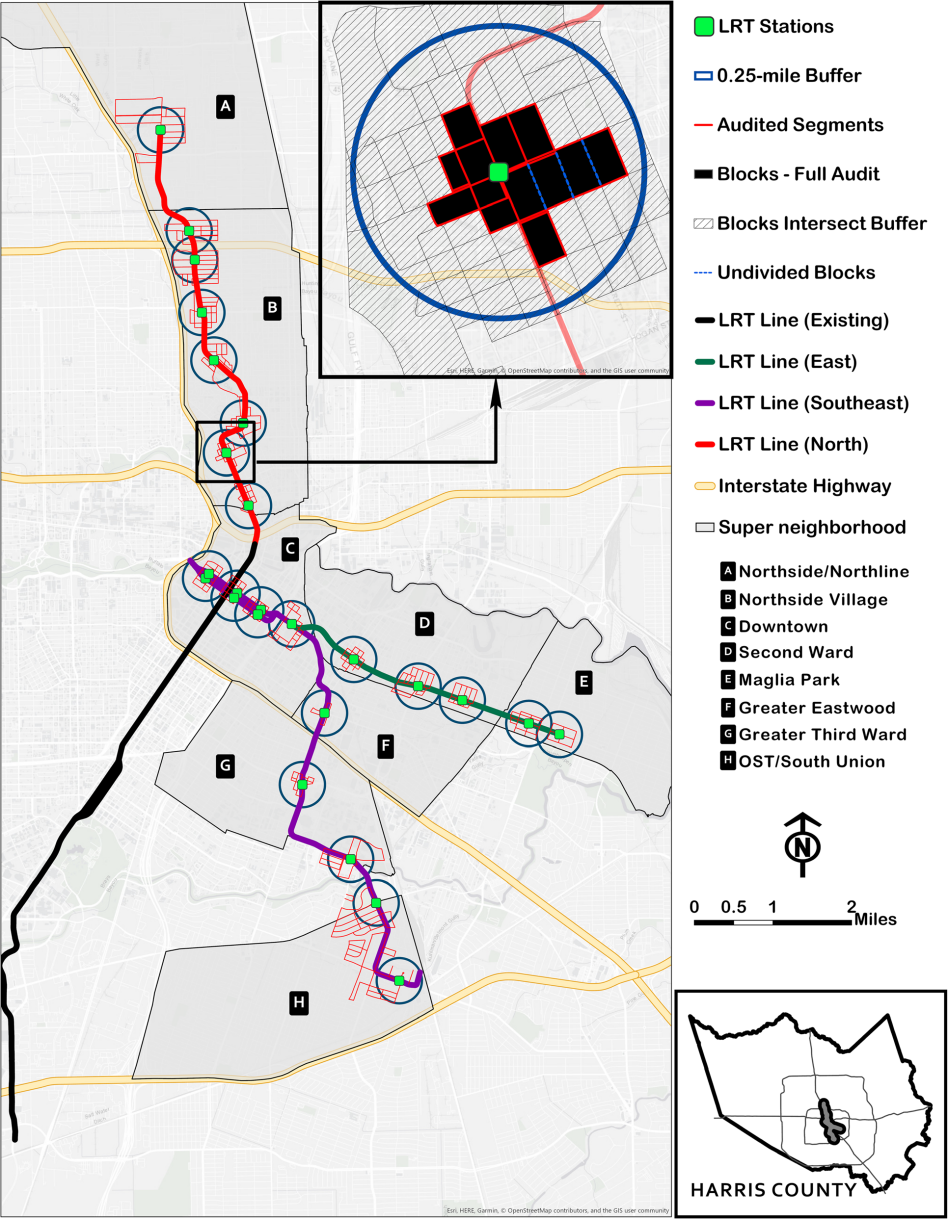
Map of the City of Houston “inner loop” showing the new light rail transit (LRT) stations that were included in the Houston TRAIN Study environmental audit exercise

To start, we chose all US Census blocks that fell, at least partially, inside the 0.25-mile LRT stations’ buffers as our sampling universe (*n* = 892 census blocks). Thus, on the map (before field visit), we referred to an individual block as an “audit block.” For our purposes, an audit block was an area that was bounded by three or more road segments. The segment for a given block was the block-facing feature on any street that also acted as a boundary for the block. Figure 1 shows a typical set of segments surrounding two adjacent blocks. In general, the rule of thumb for our audit block definition was practicable, in certain cases, however, road configurations disallowed the application of this definition rule. It is also noteworthy that although most of the blocks that were eventually audited aligned with US Census blocks, some of them did not. Two exceptions were found. First, two or more adjoining US Census blocks were not separated by streets, thereby violating our “audit block” definition (see Fig. 2 for “undivided blocks”). The second exception was that a single US Census block may have been separated into multiple “audit blocks” by intersecting streets. By protocol, all audit blocks were audited on a clock-wise path with auditors starting at the northeast corner of the block.

Prior to each day of data collection, the blocks and segments to be audited were selected and specified to the field auditors. The criteria for selection were, 1) the blocks immediately adjacent to the transit station, and 2) the maximum number of blocks which could realistically be audited based on available resources (i.e., person-hours required to audit a block) and other day-to-day circumstances on the ground (e.g., time of the day, changes in auditors’ schedules, neighborhood outlook, and weather). Finally, in certain instances, the field auditors were not able to audit a block for reasons that were not apparent in viewing the map before entering the field. For instance, some segments were inaccessible (e.g., streets inside school campuses, inaccessible rail lines running on segment, streets running inside large shopping malls, etc.) yet this was only apparent once in the field.

To provide context that will be relevant to city government and future research efforts, we organized our audit exercise efforts and data assembly while paying attention to the City of Houston (COH) “super neighborhood” boundaries. The COH Department of Neighborhoods facilitates an initiative that allows residents, civic organizations, institutions and businesses to work together to address the needs and concerns of their community within the confine of a super neighborhood; a geographically designated area—led by a council of area residents and stakeholders—that group together contiguous communities that share common physical characteristics, identity or infrastructure [26]. Houston had 88 super neighborhoods when this analysis occurred.

### Data collection, entry, and cleaning

The environmental audit was completed over eight weeks, between May 19, 2014 and July 14, 2014. Throughout the audit implementation period, a TRAIN Study co-investigator maintained oversight on all the audit activities. Two auditors with supervisory roles managed the day-to-day tasks of the auditors and provided feedback to the lead co-investigator on days that audits were conducted. For the first three weeks, auditors recorded data using paper and pencil due to technological delays. For the rest of the audit period, data were recorded electronically on tablet computers. When paper and pencil were used, all instruments were returned to TRAIN Study research staff at the end of the audit day. These data were later entered into an electronic database in Qualtrics (Qualtrics, Provo, UT). For real-time electronic data collection, we programmed the audit tool questions into Qualtrics and presented the instrument on electronic tablets. Data were saved on the local drive while auditors were on the field. At the end of every audit day, all tablets were returned to the research office. The staff connected the devices to the internet and uploaded the saved data to a dedicated Qualtrics server. After data were uploaded to the database, we completed a quality control (QC) session to confirm data completeness with all the auditors.

### Data processing and analysis – The composite index

First, we produced frequency distributions of all the features that were observed during the audit exercise. Additionally, we computed several composite index (C.I.) scores in order to produce a coherent assessment of our study area by built environment domains (e.g. land use, transportation). To better understand the surroundings of the light rail transit stops in terms of their potential influence on physical activity, we arranged a set of observed features into two classes: positive and negative. This classification was based on the expected direction of the relationship between the selected features and physical activity behavior, as evidenced by findings in available literature. For example, active commuting is more likely when walking and/or biking infrastructure are present in connected areas with varied destinations, but less likely when there are safety concerns in the built environment [27–30].

For each domain and classification, we dichotomized the observations for selected features, such that features with multiple categories were collapsed into “any observation” versus “no observation.” Thereafter, we calculated the percentage of the audited segments in the entire study area with “any observation” of these selected features. We then converted the calculated percentage to descriptive statements that ranged from “hardly ever” to “always”. Features that were observed in 0 to 10.9% of the audited segments were labeled as “hardly ever,” 11 to 30.9% were “rarely,” 31 to 45.9% were “occasionally,” and so on. We followed three steps to compute the C.I. scores. First, we assigned scores from 1.00 to 7.00 to each feature based on their label, where “hardly ever” = 1.00, “rarely” = 2.00, “occasionally” = 3.00, and so on. Second, we summed the scores for the features that are listed within each classification for each domain (e.g., scores for all the features within the positive classification in the transportation domain). Third, the calculated sum was then divided by the number of features present within the said classification, essentially computing the average score and maintaining the range for possible C.I. scores at minimum = 1.00 and maximum = 7.00. Where features have positive relationships with physical activity, a high C.I. score is indicative of prevalence of physical activity friendly features, whereas, for negative relationships, a high score indicates prevalence of features that are not physical activity friendly. The protocol that we used to calculate the C.I. scores is original. It will also be used for our follow-up audit data.

## Results

### *The study area and* key census characteristics

A map detailing the light rail transit system in Houston is found in Fig. 2. Out of the 22 stations included in the study, eight were located in the north corridor, five in the east corridor, and nine in the southeast corridor. Across all corridors, a total of 590 distinct segments were audited. Of these, 513 segments (86.9% of 590) were either completely or partially located inside the 0.25-mile buffers around the LRT stations, while 77 segments were located just outside the buffers. The 513 audited segments that intersected the buffers represented 35.7% of all accessible segments (*n* = 1439) that intersected the stations’ buffers. Overall, a total of 218 census blocks were completely surrounded by audited segments, i.e. fully audited, representing 24.4% of all the census blocks that intersected stations’ buffers (*n* = 892).The 22 new stations were located inside eight super neighborhoods, i.e., 9.1% of Houston’s 88 super neighborhoods. The distributions of the audited segments were similar in the north (41.0%) and southeast (39.7%) corridors, but much less in the east corridor (19.3%). The most audited community was the Northside Village super neighborhood (36.3%), followed by Downtown (20.2%). More data on the distributions of audited segments are shown in Table 1. Key census characteristics on audited blocks (US Census American Community Survey (ACS) 2011–2015) are presented in Table 2. Of note, audited blocks contained 11,500 people living in 5019 housing units; approximately four out of five workers drove or carpooled to work; about one out of four people earned income below the federal poverty level; and one-unit housing structures were the most common type of dwelling (57.1%).

**Table 1:** Distribution of audited segments and census blocks across LRT corridors, super neighborhoods^a^, and LRT stations. Houston TRAIN Study, 2014

LRT Corridors			Super neighborhoods^a^			Stations		
	Segment	Block	Name	Segment	Block	Name	Segment	Block
	*N* (%)	*N* (%)		*N* (%)	*N* (%)		*N* (%)	*N* (%)
North	242 (41.0)	85 (39.0)	Northside/Northline	28 (4.7)	8 (3.7)	Northline	28 (4.7)	8 (3.7)
			Northside Village	214 (36.3)	77 (35.3)	Melbourne	26 (4.4)	9 (4.1)
						Lindale Park	34 (5.8)	12 (5.5)
						Cavalcade	36 (6.1)	13 (6.0)
						Moody Park	33 (5.6)	13 (6.0)
						Fulton	26 (4.4)	10 (4.6)
						Quitman	32 (5.4)	10 (4.6)
						Burnett	27 (4.6)	10 (4.6)
East End	114 (19.3)	45 (20.6)	Magnolia Park	36 (6.1)	14 (6.4)	Magnolia	11 (1.9)	4 (1.8)
						Cesar Chavez	25 (4.2)	10 (4.6)
			Second Ward	78 (13.2)	31 (14.2)	Altic	22 (3.7)	8 (3.7)
						Lockwood	25 (4.2)	11 (5.0)
						York	31 (5.3)	12 (5.5)
Southeast	234 (39.7)	88 (40.4)	OST / South Union	60 (10.2)	24 (11)	Palm Center	35 (5.9)	13 (6.0)
						MacGregor	25 (4.2)	11 (5.0)
			Greater Third Ward	42 (7.1)	16 (7.3)	East University	20 (3.4)	9 (4.1)
						Elgin	22 (3.7)	7 (3.2)
			Greater Eastwood	14 (2.4)	4 (1.8)	Leeland	14 (2.4)	4 (1.8)
			Downtown	118 (20.0)	44 (20.2)	Bastrop	29 (4.9)	10 (4.6)
						Crawford	29 (4.9)	11 (5.0)
						Fannin	31 (5.3)	12 (5.5)
						Smith	29 (4.9)	11 (5.0)

Abbreviations: *LRT* Light Rail Transit, *TRAIN* Travel Related Activity in Neighborhoods

^a^A super neighborhood is a geographically designated area that contains contiguous communities that share common physical characteristics, identity or infrastructure. These are City of Houston signature “neighborhoods”

**Table 2:** Characteristics^a,b^ of the audited census blocks^c^ located inside each super neighborhood^d^. Houston TRAIN Study, 2014

Block characteristics	All audited blocks	Super neighborhoods^d^ (N)^e^							
		Northside/Northline	Northside Village	Second Ward	Magnolia Park	Downtown	Greater Eastwood	Greater Third Ward	OST/South Union
		(*N* = 8)	(*N* = 77)	(*N* = 31)	(*N* = 14)	(*N* = 44)	(*N* = 4)	(*N* = 16)	(*N* = 24)
Basic Profile									
Total Population (N)	11,501	1728	4895	1174	277	1091	40	608	1688
Total Housing Units (N)	5019	687	1806	424	141	1024	20	262	655
Family Households (%)	(53.0)	(55.3)	(66.3)	(61.5)	(48.7)	(32.8)	(41.2)	(46.7)	(71.2)
Nonfamily Households (%)	(47.0)	(44.7)	(33.7)	(38.5)	(51.3)	(67.2)	(58.8)	(53.3)	(28.8)
Owner Households (%)	(39.4)	(32.5)	(43.5)	(41.3)	(8.0)	(42.0)	(58.8)	(33.1)	(56.0)
Renter Households (%)	(60.6)	(67.3)	(56.5)	(58.7)	(92.0)	(58.0)	(41.2)	(66.9)	(44.0)
Below poverty level (%)	(27.4)	(36.4)	(33.0)	(30.7)	(38.6)	(15.5)	(10.0)	(35.5)	(19.1)
100–184% poverty level (%)	(18.9)	(23.9)	(20.1)	(24.3)	(24.6)	(8.7)	(7.5)	(18.8)	(23.1)
185% (plus) poverty level (%)	(53.9)	(39.7)	(46.9)	(45.0)	(37.2)	(75.9)	(82.5)	(45.9)	(57.9)
Workers Commute									
Workers Age 16+ (N)	5170	586	2214	488	106	785	27	277	687
Drove Alone to Work (%)	(72.3)	(71.3)	(69.4)	(68.9)	(84.9)	(65.1)	(77.8)	(69.0)	(71.6)
Carpooled (%)	(10.4)	(17.1)	(13.8)	(20.1)	(7.6)	(4.1)	(11.1)	(3.6)	(5.5)
Public Transportation (%)	(6.5)	(6.5)	(8.4)	(4.3)	(2.8)	(9.3)	(3.7)	(11.6)	(5.2)
Bicycled (%)	(0.5)	(0.0)	(0.2)	(1.6)	(0.0)	(1.5)	(0.0)	(0.0)	(0.4)
Walked (%)	(4.4)	(0.0)	(5.0)	(2.3)	(2.8)	(12.2)	(3.7)	(9.4)	(0.0)
OtherTransport Means (%)	(1.7)	(0.5)	(0.7)	(1.2)	(1.9)	(0.5)	(0.0)	(1.1)	(7.4)
Worked at Home (%)	(4.5)	(4.4)	(2.6)	(1.4)	(0.0)	(7.3)	(7.4)	(5.8)	(7.4)
Housing Structure									
1 Unit in Structure (%)	(57.1)	(40.8)	(70.7)	(61.8)	(32.6)	(27.3)	(85.0)	(56.1)	(82.3)
2 or 4 Units in Structure (%)	(8.8)	(3.2)	(10.9)	(21.5)	(12.1)	(3.9)	(5.0)	(13.0)	(1.1)
5 or 19 Units in Structure (%)	(11.1)	(20.7)	(13.8)	(7.6)	(29.1)	(1.6)	(5.0)	(7.3)	(3.4)
20+ Units in Structure (%)	(23.1)	(33.3)	(4.6)	(8.7)	(26.2)	(67.1)	(10.0)	(23.3)	(11.3)
Mobile Homes (%)	(0.6)	(2.0)	(0.2)	(0.5)	(0.0)	(0.0)	(0.0)	(0.0)	(2.1)

Abbreviations: *TRAIN* Travel Related Activity in Neighborhoods

^a^Data were obtained from the US Census American Community Survey (ACS) 2011–2015 estimates

^b^Selected census characteristics were those that provided quick sociodemographic outlook, insights into commuting patterns, and housing types of the immediate surroundings of the light rail transit (LRT) stations. These are characteristics of significant interest for longitudinal research

^c^Audited blocks refer to the US Census blocks that were completely surrounded by audited segments (on all sides)

^d^A super neighborhood is a geographically designated area that contains contiguous communities that share common physical characteristics, identity or infrastructure. These are City of Houston signature “neighborhoods”

^e^The number of US Census blocks in each super neighborhood

### Descriptive analysis

The characteristics of the observed features on all audited segments were organized by domains (land use, transportation, etc.). The frequency distributions of select features are shown in Table 3. An extensive list (*n* = 71) of observed features from the audit exercise is presented in several tables that are provided as supplemental materials (Additional file 1).

**Table 3:** Frequency distributions of select built environment features. Houston TRAIN Study, 2014

FEATURES	COUNT	PERCENT
A. Land Use characteristics		
Integration: Residential/Non-residential		
No Integration	425	(72.0)
A little integration	115	(19.5)
Some integration	34	(5.8)
A lot of integration	11	(1.9)
Missing data	5	(0.8)
Park		
None	565	(95.8)
One-to-Two	25	(4.3)
Playground		
None	565	(95.8)
One-to-Two	25	(4.3)
Abandoned building vacant lot		
None	329	(55.8)
One	144	(24.4)
Two	62	(10.5)
Three-to-Four	45	(7.6)
Five-to-Nine	11	(1.8)
B. Transportation characteristics		
Alternative transportation visible		
No availability	138	(23.4)
A little availability	375	(63.6)
Some availability	76	(12.9)
A lot of availability	1	(0.2)
Presence of sidewalks		
None	136	(23.1)
One side of the street	93	(15.6)
Both sides of the street	361	(61.2)
Presence of bus or other transit stops		
None	469	(79.5)
Bus stop	90	(15.3)
Other transit stop	11	(1.9)
Multiple forms of transit	20	(3.4)
Presence of bus or other transit stops		
None	469	(79.5)
Bus stop	90	(15.3)
Other transit stop	11	(1.9)
Multiple forms of transit	20	(3.4)
Traffic calming devices to reduce volume/speed		
None	115	(19.5)
A little	415	(70.3)
Some	56	(9.5)
A lot	4	(0.7)
Crossing aids for pedestrians/bicyclists		
None	200	(33.9)
A little	288	(48.8)
Some	99	(16.8)
A lot	3	(0.5)
C. Facilities characteristics		
Availability of public/recreational facilities		
No availability	540	(91.5)
A little availability	33	(5.6)
Some availability	12	(2.0)
A lot of availability	5	(0.8)
Availability of public/recreational equipment		
No availability	554	(93.9)
A little availability	22	(3.7)
Some availability	12	(2.0)
A lot of availability	2	(0.3)
D. Aesthetics characteristics		
Comfort features		
No comfort features	102	(17.3)
A few comfort features	448	(75.9)
Some comfort features	34	(5.8)
A lot of comfort features	6	(1.0)
Physical disorder visible		
No physical disorder	120	(20.3)
A little physical disorder	297	(50.3)
Some physical disorder	113	(19.2)
A lot of physical disorder	60	(10.2)
E. Signage characteristics		
“Share the road” sign		
None	567	(96.1)
A Few (1–3)	22	(3.7)
Some (4–6)	0	(0.0)
A Lot (&gt;7)	1	(0.2)
Other pedestrian or bicyclist friendly traffic sign		
None	275	(46.6)
A Few (1–3)	292	(49.5)
Some (4–6)	21	(3.6)
A Lot (7+)	2	(0.3)
F. Social environment		
People visible in this segment		
None	140	(23.7)
A Few (1–3)	261	(44.2)
Some (4–6)	77	(13.1)
A Lot (7+)	112	(19.0)
Teenagers or adults engaging in active behaviors		
None	214	(36.3)
A Few (1–3)	241	(40.8)
Some (4–6)	62	(10.5)
A Lot (7+)	73	(12.4)

Abbreviations: *TRAIN* Travel Related Activity in Neighborhoods

In terms of land use in the areas surrounding the LRT stations, 72% segments had no integration between residential and non-residential use and 44.2% included one or more abandoned building or vacant lot. Only 4.3% of segments had a park while another 4.3% had a playground (either at a park or within a school). Active transportation infrastructure (e.g. for pedestrians, bicyclists, or public transit users) were not available at all in 23.4% of the segments while they were “a little available” in 63.6%. Sidewalks were present in either one or both sides of the street in 76.9% of segments and 20.6% included a bus or other transit stop. Traffic calming devices (e.g., speed humps and traffic signals) and crossing aids (e.g., crosswalks and traffic lights) were present in 80.5 and 66.1% of the segments, respectively. A small proportion of the audited segments had public/recreational facilities (8.7%) and public/recreational equipment (6.5%). Comfort features (shade trees, benches etc.) were present “some” and “a lot” in 5.8 and 1.0% of the segments respectively, but not present at all in 17.3%. Physical disorder (i.e. litter, rubbish, graffiti, broken glass, etc.) was visible “some” or “a lot” in 29.4% of the segments. Pedestrian or bicyclist friendly traffic signs were the most prevalent signage in the study area, appearing “a few” times in 49.5% of the segments, although signs that specifically listed “share the road” were much less present (3.9%). Of the 76.3% of the study segments with people visible in the street, 63.7% of those had teenagers and adults that were engaged in some type of physical activity (walking, biking, playing sport, etc.).

### Qualitative description and composite index

To better appreciate the built environment around the light rail transit stations as it related to physical activity, we transposed observed data on selected features into qualitative descriptions based on positive vs. negative relationship with physical activity (Table 4). For land use, all 8 positive features were either “hardly ever” or “rarely” present, while 2 out of 6 negative features were between “half the time” and “usually” present. For transportation, 6 out of 18 positive features were either “hardly ever” or “rarely” present, 10 were either “frequently” or “usually” present, and one feature, street lighting, was “always” present. One of 2 negative features, damaged sidewalks, was “occasionally” present, and the second was “rarely” present. For facilities, 3 out of 3 features, all positive, were “hardly ever” present. For aesthetics, 2 out of 2 positive features were either “frequently” or “usually” present, while 3 out of 6 negative features were “usually” present. For the remaining domains, the number of positive features that were between “hardly ever” present and “rarely” present were: 4 out of 5 for signage; and 4 out of 6 for social environment. Negative features were 1 “rarely” present and 1 “occasionally” present for signage, and 1 “rarely” present for social environment. The composite index scores (min = 1, max = 7) for: land use were 1.25 for positive classification (P) and 3.00 for negative classification (N); transportation were 4.06 (P) and 2.50 (N); facilities was 1.00 (P); aesthetics were 5.50 (P) and 4.17 (N), signage were 1.60 (P) and 2.50 (N); and social environment were 2.83 (P) and 2.00 (N).

**Table 4:** Calculating the Composite Index (C.I.) scores for the six domains that were represented in the environmental audit instrument. Data presented are for the entire study area (590 segments). Houston TRAIN Study, 2014

Descriptive statements			Hardly ever	Rarely	Occasionally	Half the time	Frequently	Usually	Always	Composite Index^b^	
Percent ranges			0–10	11–30	31–45	46–55	56–70	71–90	91–100		
Scores			1.0	2.0	3.0	4.0	5.0	6.0	7.0		
	P/N^a^	(%)									
Land Use											
Land use integration	P	27.1		2						1.25	(P)
Office building	P	10.3	1								
Other services – e.g. beautician and lawyer	P	12.5		2							
Fast food restaurants	P	8.6	1								
Strip mall	P	5.9	1								
Transportation facility	P	6.8	1								
Place of worship	P	6.1	1								
Park	P	4.2	1								
Warehouses, factories, or industrial buildings	N	14.1		2						3.00	(N)
Auto shop	N	10.2	1								
Parking lot or parking garage	N	50.3				4					
Driveway (either residential or non-residential)	N	89.5						6			
Abandoned building or vacant lot	N	44.2			3						
Major transportation development (e.g. bridge, tunnel)	N	14.2		2							
Transportation											
Any alternative transportation modes	P	76.6						6		4.06	(P)
Any sidewalk facility	P	76.9						6			
Sidewalk continuity; at least 1 side of street (both ends)^c^	P	67.0					5				
Curvilinear curbs; at least 1 side of street (both ends)^c^	P	60.1					5				
Sidewalk coverage on left side of segment (> 75%)^c^	P	74.9						6			
Sidewalk coverage on right side of segment (> 75%)^c^	P	70.9					5				
Bike lane or marked shoulder (for bikes)	P	2.7	1								
Bike racks present	P	2.5	1								
Bus/transit stop present	P	20.5		2							
Bus stop; covered shelter with/out bench^c^	P	60.3					5				
Non-concrete multi-use trails or paths	P	4.4	1								
Posted speed limit	P	7.1	1								
On-street parking	P	36.8			3						
> 3 directions at intersections	P	88.6						6			
Road design to reduce car volume/speed	P	17.8		2							
Traffic calming devices to reduce car volume/speed	P	80.5						6			
Crossing aids for pedestrian / bicyclist	P	66.1					5				
Street lighting for sidewalk, street shoulders, etc.	P	96.1							7		
Sidewalk has heaves, cracks, broken sections, etc.^c^	N	34.8			3					2.50	(N)
Sidewalk blocked by obstacles^c^	N	28.2		2							
Facilities											
Public / recreational facilities	P	8.5	1							1.00	(P)
Public / recreational equipment	P	6.1	1								
Playground equipment	P	3.9	1								
Aesthetics											
Attractive features (e.g., architecture, vegetation)	P	62.0					5			5.50	(P)
Comfort features (shade, trees, benches, etc.)	P	82.9						6			
Air pollution	N	9.2	1							4.17	(N)
Noise pollution	N	38.3			3						
Physical disorder (general)	N	79.7						6			
Whole or broken beer or liquor bottles or can	N	42.9			3						
Cigarette, cigar butts or discarded cigarette packages	N	71.0						6			
Garbage, litter, or broken glass	N	73.7						6			
Signage											
Pedestrian or bicyclist friendly traffic signs	P	53.4				4				1.60	(P)
Share the road sign	P	3.9	1								
Religious message	P	8.1	1								
Political message	P	5.9	1								
Fast food billboard	P	6.6	1								
No trespassing / beware of dogs	N	33.2			3					2.50	(N)
SSecurity warning	N	15.3		2							
Social Environment											
Children present in the street	P	7.3	1							2.83	(P)
Children engaged in active behavior	P	5.3	1								
Teenagers/adults present in the street	P	72.9						6			
Teenagers/adults engaged in active behavior	P	63.9					5				
Older adults (> 65 years) present in the street	P	13.1		2							
Older adults (> 65 years) engaged in active behavior	P	12.2		2							
Stray dogs or animals (not squirrels or rabbits)	N	11.5		2						2.00	(N)

Abbreviations: *TRAIN*, Travel Related Activity in Neighborhoods

^a^Observed features were arranged into two classes: positive (P) and negative (N). This classification was based on the expected direction of the relationship between each feature and physical activity behavior, as evidenced by findings in available literature

^b^C.I. scores were calculated separately for each class (positive vs. negative) in each domain. To calculate a C.I. score, we first dichotomized the observed categories for selected features, such that features with multiple categories were collapsed into “any observation” versus “no observation”. Thereafter, we calculated the percentage of all the audited segments in the entire study area with “any observation” of these selected features. We then converted the calculated percentage to seven descriptive statements that ranged from “hardly ever” to “always”. For example, a feature that was observed in 0–10.9% of the audited segments was labeled “hardly ever,” and so on. The seven descriptive statements were thereafter assigned scores, ranging from 1.00 for “hardly ever” (0–10.9%) to 7.00 for “always” (91–100%). We then calculated the average score for that class and maintained the min = 1.00, max = 7.00 average score (i.e., the C.I. score). Where positive relationship with physical activity is expected, a high C.I. score is indicative of prevalence of physical activity friendly features, whereas, for negative relationships, a high score indicates prevalence non-physical activity friendly features

^c^Data shown (percentages) are based on a subset of the audited 590 segments that are applicable to these particular audit questions. For example. Sidewalk continuity only applies to segments with sidewalks

Table 5 shows the composite index (C.I.) scores for each super neighborhood. Land use C.I. scores across all super neighborhoods were less than 2.00 for the positive classification, except for Northside/Northline super neighborhood, while C.I. scores were higher than 3.00 for negative classification in all super neighborhoods except one. For transportation, C.I. scores for positive classification ranged from 3.72 to 4.67; Downtown super neighborhood had the highest score. Transportation negative classification C.I. scores were lower than 2.50 for five super neighborhoods, while the C.I. score for Magnolia Park was 4.00. The facilities domain, which only has positive classification, had low C.I. scores across all super neighborhoods (min = 1.00; max = 1.67). The C.I. scores for aesthetics were generally high for both positive (min = 4.00; max = 6.00) and negative (min = 3.67; max = 4.83) classifications. Downtown had the highest C.I. score for positive classification. The C.I. scores for signage and social environment domains were generally low for both positive and negative classifications. For signage, the minimum and maximum C.I. scores were 1.40 and 2.00 respectively for positive, and 1.50 and 3.50 respectively for negative classification. The C.I. scores in the social environment domain were generally higher for positive classification (min = 2.17; max = 3.17) than for negative classification where the scores for all but one super neighborhood were 2.00 or less. Detailed calculations of the C.I. scores for each super neighborhood are shown in several tables that are provided as supplemental materials (Additional file 2).

**Table 5:** Composite Index (C.I.) scores for each super neighborhood by built environment domains and expected relationship (P/N^a^) with physical activity. Houston TRAIN Study, 2014

	Northside/ Northline	Northside Village	Downtown	Second Ward	Magnolia Park	Greater Eastwood	Greater Third Ward	OST/ South Union	ALL
Segments *N* (%)	28 (4.75)	214 (36.27)	118 (20)	78 (13.22)	36 (6.1)	14 (2.37)	42 (7.12)	60 (10.17)	590 (100)
Land use									
Positive	2.00	1.25	1.25	1.25	1.63	1.50	1.25	1.50	1.25
Negative	3.83	3.17	3.00	3.50	3.67	3.33	2.67	3.00	3.00
Transportation									
Positive	3.72	3.72	4.67	3.83	4.06	4.17	4.06	4.06	4.06
Negative	1.00	2.50	2.00	3.50	4.00	3.50	1.50	2.00	2.50
Facilities									
Positive	1.67	1.00	1.33	1.00	1.00	1.00	1.33	1.67	1.00
Aesthetics									
Positive	5.50	5.00	6.00	5.00	4.00	5.00	5.50	5.50	5.50
Negative	4.50	3.83	4.50	4.17	4.83	4.50	4.17	3.67	4.17
Signage									
Positive	1.80	1.60	2.00	1.60	1.80	1.40	2.00	1.40	1.60
Negative	3.50	2.50	1.50	2.50	2.50	2.00	2.00	2.50	2.50
Social environment									
Positive	3.17	2.67	3.00	2.17	2.50	2.17	2.33	3.00	2.83
Negative	2.00	2.00	1.00	2.00	1.00	3.00	2.00	1.00	2.00

^a^Observed features were arranged into two classes: positive (P) and negative (N). This classification was based on the expected direction of the relationship between each feature and physical activity behavior, as evidenced by findings in available literature

## Discussion

This study described the micro-scale built environment features of the surrounding blocks of newly installed light rail transit stops in Houston. We did so by using an adapted comprehensive field audit procedure, which captures features known to be of relevance for transit use, active travel (utilitarian walking and bicycling), and physical activity behaviors at large. Overall, the results of the audit suggest the built environment surrounding the newly installed light rail stations would not be considered as highly supportive of active travel and physical activity behaviors, and thus, may contribute to suboptimal use of the new light rail transit stops.

The results showed that the audited segments lacked land use integration, were mostly residential, and almost half had at least one abandoned building or vacant lot. One quarter of the audited areas lacked a sidewalk. When available, sidewalks were rarely blocked with obstacles. However, they were occasionally uneven and not always complete. There was virtually no bicycle-friendly infrastructure, minimal presence of public/recreational facilities that would support physical activity, and considerable presence of physical disorder. Although there were few people present in the built environment audited, of those observed, the majority were teenagers or adults engaging in walking, while there were virtually no children observed. Notably, all audit sessions were completed between 9 am and 3 pm during the months of May and June. Generally, students attending Houston Independent School District (HISD) are in school during the day, and summer break usually begins at the beginning of June.

We found there to be little mixing of land use types around the transit stops, a feature which has been found to be positively associated with active travel and physical activity behaviors [31, 32]. However, past findings pertain mostly to studies examining the features of the home neighborhood environment (i.e., residential areas) as they relate to walking for transport. In these settings, it makes sense that higher land use mix, which implies a broader variety of accessible destinations, would lead to more active travel, since residents walk to these destinations from their home location. Because our study audited blocks surrounding transit stops, the interpretation of these results must be placed into context. It may be possible to promote active travel behaviors among city residents by adding new public transit infrastructure that connects their residential neighborhoods with commercial districts. This principle should hold true independently of the land-use mix of the home neighborhood, which residents can presumably leave via public transit to reach their destinations in other areas of the city.

Almost half of the audited segments had at least one abandoned building or vacant lot, there was physical disorder in over 70% of the audited areas, occasional presence of broken bottles, usually liquor bottles, and litter/garbage in the street in over almost 75% of the audited areas. It is thought that physical disorder can be an artifacts of criminal behavior or heighten residents’ fear or perceptions of crime [33–36]. That, along with social disorder (arguing/yelling, loitering, drug sales/use), of which there was little observed in the current study, are thought to negatively impact physical activity, though the literature in this area is mixed [37].

Notably, there was very little transportation infrastructure in the audited areas designed to support active travel and physical activity at large (e.g. bike lanes, multi-use walking/biking trails, playgrounds). Almost 25% of the audited areas did not have a sidewalk, which could pose a significant barrier for both recreational and utilitarian walking in these areas. This is of specific relevance to this study, focused on new light-rail stops. Without basic pedestrian infrastructure (i.e., sidewalks) to safely connect people to the transit stops, utilization of public transit could be significantly impaired.

There were, however, high proportions (66%) of the segments had crossing aids (e.g., crosswalks, stop signs, traffic lights, curb extensions), along with traffic calming devices (e.g., diverters, speed humps, traffic signals) in 80% of segments. These features are supportive of physical activity [38–40], yet not likely to have a dramatic effect on physical activity behaviors if the segments lack connectivity, mixed land use, and population density [41]. It should be noted that these features are, however, important factors to consider for pedestrian safety [42]. Also, though 96% of segments had at least one street lighting (see supplementary material A), two Houston-specific phenomena may have lessened the significance of this finding. First, there is a generous distribution of street lighting inside the city core in general [43], and recent analysis of the city’s data suggests mixed evidence at best, and possibly counter-intuitive findings on the streetlights-safety relationship [44].

To our knowledge, no previous peer-reviewed studies have specifically characterized the micro-scale built environment around newly implemented transit stops spanning a variety of areas within a city, with specific focus on the urban features known to be relevant for public transit use, active travel, and physical activity behaviors. In a 2009 study, Ryan and Frank analyzed the macro-scale built environment (land use mix, density, and street connectivity) around transit stops and found “walkability” around transit stops is significantly associated with transit ridership, in expected directions [45]. Further, some studies from international settings (Thailand, Latin America) are available measuring the built environment surrounding pre-existing transit stops and its association with travel-related behavioral outcomes [46, 47]. However, those studies also mainly focused on macro-scale aspects of the built environment, while our work examines the micro-environment. Other studies have focused on measuring livability of different transit corridors, or the local features related to active travel and transit use when reaching shopping districts specifically [48, 49]. Our work, in contrast, captures essential aspects of neighborhood walkability and activity-friendliness at large, and included transit stops across a variety of districts (residential, shopping, medical center, etc.) across the city of Houston. Although aspects related to walkability or activity-friendliness are sometimes included as a component of the broader concept of livability, these are distinct concepts. A substantial body of evidence is available demonstrating the association between walkability and active travel and physical activity behaviors [50–52]. Although the present study did not measure active travel or physical activity, the growing literature in this field suggests that these results describe a built environment not supportive of utilitarian walking or cycling, which in turn likely affects rates of public transit use in these settings [53–56].

A special consideration that should be noted when interpreting these results is the City of Houston’s lack of a general land use plan and a lack of a formal zoning policy [57]. Despite this, land use in the city is still subject to almost the same degree of regulation as in other major American cities, but these regulations are accomplished via alternative mechanisms, including: municipal ordinances neighborhood deed restrictions, municipal management districts, tax increment reinvestment zones, super neighborhoods, lawsuits and other de jure and de facto mechanisms [58]. These entities have an outsized ability to direct capital improvements, and in some cases can actually finance and implement infrastructure improvements somewhat independently of the City of Houston. To any degree that this land use governance departs from other major US cities, the external validity of our audit results may be limited.

The timing of the audits also merits consideration. The audits were conducted in the summer of 2014, which means they were done when one segment of the LRT extension was open, while the other two were not yet operational. Despite not being operational, the stations themselves and their associated infrastructure were virtually entirely complete at the time of the audit. However, because the trains were not operational at that time, pedestrian and bike traffic in the area may have been reduced compared to when the lines are active. As a result, the built environment features that are sensitive to pedestrian and bike traffic, namely features related to the social environment domain, might not be representative of the station environment as compared to when the trains are operational. Regardless, we expect that the timing had little to no influence on the other built environment features that were observed during our audit exercise.

Besides the timing of the audit relative to the LRT opening, we also note that aspects of the social and aesthetic environment (i.e. presence of people on segment; noise pollution; air pollution) could have been different had the audit been conducted at either different times of day or times of the year. This is an inherent limitation of all “point in time” observational data collection activities. Despite this limitation, we note that it is unlikely any other aspects of the audit, mainly “hard” physical infrastructure, would have been affected had they been assessed at alternative times of day or year. Also, our efforts were thorough and resource-intensive, but we could not audit all the accessible segments that intersected the 0.25-mile buffer. Though, we are unaware of any specific evidence that our 35.7% reach provided an accurate representation of the areas encompassed by the .25 buffers, we consider the one-third coverage generally acceptable. Regarding measurement considerations, the absence of inter-rater reliability tests may have introduced measurement errors during the audit, though, our real time discussions of observed values during the training session may have lessened the propensity for such errors.

## Conclusion

In conclusion, most of the areas around the new light rail transit stops in the TRAIN Study were not found to have the known features of supportive neighborhoods for active travel (utilitarian walking and cycling) and physical activity behaviors at large at baseline. However, the results of this study will be used in future analyses for assessing the short- and long-term impact of accessibility to the light rail transit stops. Future research will also examine the impact of the walkability and activity-friendliness of the micro built environment surrounding new transit stops on light rail transit use, active travel, and on total physical activity. Additionally, these data allow examination of environmental factors that possibly interact with light rail transit exposure to differentially effect transit use. Aside from the main descriptive results, this paper provides a detailed description of the methods used to conduct a field-based, micro-scale, environmental audit. The methods detail the procedures for each step in the audit, from selecting and defining the audit segments, and training auditors, to data collection, entry, cleaning and analysis. This study can be useful for future researchers and practitioners interested in conducting a similar audit of the micro-scale built environment surrounding new transit infrastructure, from the ground-up.

## Additional files

Additional file 1: Frequency distributions of select built environment features. Houston TRAIN Study, 2014. This file contains several Tables that show the frequency distributions of an extensive list of observed features (*n* = 71) from the audit exercise. (DOCX 38 kb)

Additional file 2: Calculating the Composite Index (C.I.) scores for the six domains that were represented in the environmental audit instrument. Houston TRAIN Study, 2014. This file contains several Tables that provide detailed information on how we calculated the composite index (C.I.) scores for all the six built environment domains that were represented in the environmental audit instrument. (DOCX 224 kb)

## Abbreviations

AA-Tool: Analytic Audit Tool
C.I.: Composite Index
GIS: Geographic Information System
LRT: Light Rail Transit
METRO: Metropolitan Transit Authority of Harris County
TRAIN: Travel Related Activity In Neighborhoods
