# Supplementary material for: Foot-based audit of streets adjacent to new light rail stations in Houston, Texas: measurement of health-related characteristics of the built environment for physical activity research
Source: BMC Public Health. 2019 Feb 28;19:238. doi: 10.1186/s12889-019-6560-4 (PMC6393971; doi:10.1186/s12889-019-6560-4)
Supplement: Supplementary file 1 — Frequency distributions of select built environment features. Houston TRAIN Study, 2014. This file contains several Tables that show the frequency distributions of an extensive list of observed features (n = 71) from the audit exercise. (DOCX 38 kb) [file 12889_2019_6560_MOESM1_ESM.docx]

**TITLE: Foot-based audit of streets adjacent to new light rail stations in Houston, Texas: Measurement of health-related characteristics of the built environment for physical activity research.**

**Supplemental Material (A): Frequency distributions of select built environment features. TRAIN Study, 2014**

**Table A1.** Frequency distributions of select **land use** features, TRAIN Study, 2014.

| **Land use types and characteristics** | **Count** | **Percent** |
| --- | --- | --- |
| **A. Residential Land use** |  |  |
| **Integration: Residential/Non-residential** |  |  |
| No Integration | 425 | (72.0) |
| A little integration | 115 | (19.5) |
| Some integration | 34 | (5.8) |
| A lot of integration | 11 | (1.9) |
| Missing data | 5 | (0.8) |
|  |  |  |
| **Single-family home present** |  |  |
| None | 273 | (46.3) |
| One-to-Five | 162 | (27.5) |
| Six-to-Ten | 61 | (10.3) |
| 11-15 | 19 | (3.2) |
| 16-20 | 27 | (4.6) |
| 21-30 | 20 | (3.4) |
| 31 and above | 28 | (4.7) |
|  |  |  |
| **Two- to six-family structure** |  |  |
| None | 559 | (94.7) |
| One-to-Two | 26 | (4.4) |
| Three-to-Five | 5 | (0.9) |
|  |  |  |
| **Apartment/complex or condominium** |  |  |
| None | 552 | (93.6) |
| One-to-Two | 32 | (5.4) |
| Three-to-Eight | 5 | (0.9) |
| Twenty | 1 | (0.2) |
|  |  |  |
| **Any residential land use on segment** |  |  |
| No residential land use | 218 | (36.9) |
| Some type of residential land use | 372 | (63.1) |
|  |  |  |
| **B. Commercial Land use** |  |  |
| **Fast food restaurant** |  |  |
| None | 539 | (91.4) |
| One-to-Two | 48 | (8.1) |
| Three-to-Eight | 3 | (0.5) |
|  |  |  |
| **Strip mall or shopping center** |  |  |
| None | 555 | (94.1) |
| One-to-Two | 32 | (4.4) |
| Three-to-Five | 3 | (0.5) |
|  |  |  |
| **Warehouses, factories, industrial buildings** |  |  |
| None | 507 | (85.9) |
| One-to-Two | 73 | (12.4) |
| Three-to-Five | 10 | (1.7) |
|  |  |  |
| **Office building** |  |  |
| None | 529 | (89.7) |
| One-to-Two | 52 | (8.9) |
| Three-to-Ten | 9 | (1.5) |
|  |  |  |
| **Auto shop** |  |  |
| None | 530 | (89.8) |
| One-to-Two | 55 | (9.3) |
| Three-to-Five | 5 | (0.8) |
|  |  |  |
| **Other services (beautician, lawyer)** |  |  |
| None | 514 | (87.1) |
| One-to-Two | 64 | (10.9) |
| Three-to-Five | 8 | (1.3) |
| Six-to-Seven | 2 | (0.4) |
| Missing | 2 | (0.3) |
|  |  |  |
| **C. Public Land use** |  |  |
| **Place of worship** |  |  |
| None | 554 | (93.9) |
| One-to-Three | 36 | (6.1) |
|  |  |  |
| **Junior college, college/university** |  |  |
| None | 576 | (97.6) |
| One-to-Two | 12 | (2.0) |
| Three-to-Four | 2 | (0.4) |
|  |  |  |
| **Transportation facility (transit station)** |  |  |
| None | 550 | (93.2) |
| One-to-Two | 34 | (5.8) |
| Three-to-Four | 6 | (1.0) |
|  |  |  |
| **Other (courthouse, utilities, etc.)** |  |  |
| None | 564 | (95.6) |
| One-to-Three | 26 | (4.4) |
|  |  |  |
| **D. Recreational Land use** |  |  |
| **Indoor fitness facility** |  |  |
| None | 579 | (98.1) |
| One-to-Two | 11 | (1.9) |
|  |  |  |
| **Park** |  |  |
| None | 565 | (95.8) |
| One-to-Two | 25 | (4.3) |
|  |  |  |
| **Playground** |  |  |
| None | 565 | (95.8) |
| One-to-Two | 25 | (4.3) |
|  |  |  |
| **Sports/playing field** |  |  |
| None | 571 | (96.8) |
| One-to-Two | 17 | (2.9) |
| Three-to-Five | 2 | (0.4) |
|  |  |  |
| **E. Other Destinations** |  |  |
| **Parking lot or parking garage** |  |  |
| None | 293 | (49.7) |
| One | 153 | (25.9) |
| Two | 86 | (14.6) |
| Three | 29 | (4.9) |
| Four-to-Ten | 27 | (4.7) |
| Twenty-one | 2 | (0.3) |
|  |  |  |
| **Driveway** |  |  |
| None | 62 | (10.5) |
| One-to-Five | 311 | (52.7) |
| Six-to-Ten | 92 | (15.6) |
| 11-15 | 41 | (6.9) |
| 16 - 20 | 29 | (4.9) |
| 21 - 30 | 24 | (4.1) |
| 31 - 40 | 25 | (4.2) |
| 41 - 55 | 6 | (1.0) |
|  |  |  |
| **Abandoned building vacant lot** |  |  |
| None | 329 | (55.8) |
| One | 144 | (24.4) |
| Two | 62 | (10.5) |
| Three-to-Four | 45 | (7.6) |
| Five-to-Nine | 11 | (1.8) |
|  |  |  |
| **Highway, tunnel, bridge, railroad** |  |  |
| None | 506 | (85.8) |
| One | 75 | (12.7) |
| Two-to-Three | 9 | (1.6) |

**Table A2.** Frequency distributions of select **transportation** features, TRAIN Study, 2014.

| **Transportation characteristics** | **Count** | **Percent** |
| --- | --- | --- |
| **A. Transportation environment** |  |  |
| **Alternative transportation visible** |  |  |
| No availability | 138 | (23.4) |
| A little availability | 375 | (63.6) |
| Some availability | 76 | (12.9) |
| A lot of availability | 1 | (0.2) |
|  |  |  |
| **B. Sidewalks** |  |  |
| **Presence of sidewalks** |  |  |
| None | 136 | (23.1) |
| One side of the street | 93 | (15.6) |
| Both sides of the street | 361 | (61.2) |
|  |  |  |
| **Buffer** |  |  |
| Adjacent to street or curb (no buffer) | 124 | (21.0) |
| Within 2 ft of street buffer | 37 | (6.3) |
| Between 2 and 6 ft of the street buffer | 160 | (27.1) |
| Greater than 6 ft of street buffer | 133 | (22.5) |
| Does not apply | 136 | (22.7) |
|  |  |  |
| **Continuity of sidewalks** |  |  |
| Not continuous | 42 | (7.1) |
| Continuous at one end | 108 | (18.3) |
| Continuous at both ends | 304 | (51.5) |
| Does not apply | 136 | (23.1) |
|  |  |  |
| **Sidewalk width** |  |  |
| 0 to 3 ft | 34 | (5.8) |
| >3 ft and | 334 | (56.6) |
| >6 ft | 86 | (14.6) |
| Does not apply | 136 | (23.1) |
|  |  |  |
| **Levelness and condition of sidewalk** |  |  |
| None | 296 | (50.1) |
| A little | 107 | (18.1) |
| Some | 37 | (6.3) |
| A lot | 14 | (2.4) |
| Does not apply | 136 | (23.1) |
|  |  |  |
| **Obstructions** |  |  |
| None | 326 | (55.2) |
| A little | 78 | (13.2) |
| Some | 32 | (5.4) |
| A lot | 18 | (3.1) |
| Does not apply | 136 | (23.1) |
|  |  |  |
| **Curvilinear curbs** |  |  |
| None | 89 | (15.0) |
| On only one end | 92 | (15.6) |
| On both ends | 42 | (7.1) |
| On both sides and ends | 231 | (39.2) |
| Does not apply | 136 | (23.1) |
|  |  |  |
| **Percent of sidewalk coverage for left side** |  |  |
| 0-25% | 81 | (13.7) |
| 26-50% | 17 | (2.9) |
| 51-75% | 16 | (2.7) |
| 76-100% | 340 | (57.6) |
| Does not apply | 136 | (23.1) |
|  |  |  |
| **Percent of sidewalk coverage for right side** |  |  |
| 0-25% | 104 | (17.6) |
| 26-50% | 17 | (2.9) |
| 51-75% | 11 | (1.8) |
| 76-100% | 322 | (54.6) |
| Does not apply | 136 | (23.1) |
|  |  |  |
| **C. Bikelanes** |  |  |
| **Presence of bikelanes** |  |  |
| None | 572 | (96.9) |
| One side of the street | 7 | (1.2) |
| Both sides of the street | 9 | (1.5) |
| Missing data | 2 | (0.3) |
|  |  |  |
| **Presence of Bike racks** |  |  |
| None | 575 | (97.5) |
| One side of the street | 13 | (2.2) |
| Both sides of the street | 2 | (0.3) |
|  |  |  |
| **D. Transit** |  |  |
| **Presence of bus or other transit stops** |  |  |
| None | 469 | (79.5) |
| Bus stop | 90 | (15.3) |
| Other transit stop | 11 | (1.9) |
| Multiple forms of transit | 20 | (3.4) |
|  |  |  |
| **Presence of bench/covered shelter** |  |  |
| None | 45 | (7.6) |
| Bench | 3 | (0.5) |
| Covered shelter | 16 | (2.7) |
| Both | 57 | (9.7) |
| Does not apply | 469 | (79.5) |
|  |  |  |
| **E. Other transportation characteristics** |  |  |
| **Presence of path or trail** |  |  |
| None | 564 | (95.6) |
| One side of the street | 15 | (2.5) |
| Both sides of the street | 11 | (1.9) |
|  |  |  |
| **Posted general speed limit of:** |  |  |
| 5 | 1 | (0.2) |
| 20 | 8 | (1.4) |
| 30 | 12 | (2.0) |
| 35 | 19 | (3.2) |
| 40 | 1 | (0.2) |
| 50 | 1 | (0.2) |
| None posted | 548 | (92.9) |
|  |  |  |
| **Availability of on-street parking** |  |  |
| Yes | 217 | (36.8) |
| No | 373 | (63.2) |
|  |  |  |
| **Other street characteristics to reduce volume/speed** |  |  |
| None | 485 | (82.3) |
| A little | 97 | (16.4) |
| Some | 6 | (1.0) |
| A lot | 2 | (0.3) |
|  |  |  |
| **Traffic calming devices to reduce volume/speed** |  |  |
| None | 115 | (19.5) |
| A little | 415 | (70.3) |
| Some | 56 | (9.5) |
| A lot | 4 | (0.7) |
|  |  |  |
| **Crossing aids for pedestrians/bicyclists** |  |  |
| None | 200 | (33.9) |
| A little | 288 | (48.8) |
| Some | 99 | (16.8) |
| A lot | 3 | (0.5) |
|  |  |  |
| **Street lighting** |  |  |
| None | 25 | (4.3) |
| A little | 251 | (42.5) |
| Some | 170 | (28.8) |
| A lot | 144 | (24.4) |

**Table A3.** Frequency distributions of select **facilities** features, TRAIN Study, 2014.

| **Facilities characteristics** | **Count** | **Percent** |
| --- | --- | --- |
| **Availability of public/recreational facilities** |  |  |
| No availability | 540 | (91.5) |
| A little availability | 33 | (5.6) |
| Some availability | 12 | (2.0) |
| A lot of availability | 5 | (0.8) |
|  |  |  |
| **Availability of public/recreational equipment** |  |  |
| No availability | 554 | (93.9) |
| A little availability | 22 | (3.7) |
| Some availability | 12 | (2.0) |
| A lot of availability | 2 | (0.3) |
|  |  |  |
| **Playground equipment** |  |  |
| None | 567 | (96.1) |
| One-to-two | 13 | (2.2) |
| Three-to-four | 8 | (1.4) |
| Five or more | 2 | (0.3) |
|  |  |  |
| **Availability of service amenities: Trash bins** |  |  |
| Visible | 219 | (37.2) |
| Not Visible | 371 | (62.8) |

**Table A4.** Frequency distributions of select **aesthetics** features, TRAIN Study, 2014.

| **Aesthetics characteristics** | **Count** | **Percent** |
| --- | --- | --- |
| **Attractive features** |  |  |
| No attractive features | 226 | (38.3) |
| A few attractive features | 268 | (45.4) |
| Some attractive features | 89 | (15.1) |
| A lot of attractive features | 7 | (1.2) |
|  |  |  |
| **Comfort features** |  |  |
| No comfort features | 102 | (17.3) |
| A few comfort features | 448 | (75.9) |
| Some comfort features | 34 | (5.8) |
| A lot of comfort features | 6 | (1.0) |
|  |  |  |
| **Air pollution (seen or smelled)** |  |  |
| No air pollution | 536 | (90.8) |
| A little air pollution | 47 | (8.0) |
| Some air pollution | 6 | (1.0) |
| A lot of air pollution | 1 | (0.2) |
|  |  |  |
| **Noise pollution audible** |  |  |
| No noise pollution | 364 | (61.7) |
| A little noise pollution | 184 | (31.2) |
| Some noise pollution | 41 | (6.9) |
| A lot of noise pollution | 1 | (0.2) |
|  |  |  |
| **Physical disorder visible** |  |  |
| No physical disorder | 120 | (20.3) |
| A little physical disorder | 297 | (50.3) |
| Some physical disorder | 113 | (19.2) |
| A lot of physical disorder | 60 | (10.2) |
|  |  |  |
| **Physical disorder: broken beer or liquor bottles or cans** |  |  |
| None | 337 | (57.1) |
| A Few (1-3) | 171 | (29.0) |
| Some (4-6) | 38 | (6.4) |
| A Lot (7+) | 44 | (7.5) |
|  |  |  |
| **Physical disorder: cigarette, cigar butts, or discarded cigarette packages** |  |  |
| None | 171 | (29.0) |
| A Few (1-3) | 156 | (26.4) |
| Some (4-6) | 78 | (13.2) |
| A Lot (7+) | 185 | (31.4) |
|  |  |  |
| **Physical disorder: garbage, litter, or broken glass** |  |  |
| None | 155 | (26.3) |
| A Few (1-3) | 154 | (26.1) |
| Some (4-6) | 121 | (20.5) |
| A Lot (7+) | 160 | (27.1) |
|  |  |  |
| **Physical disorder: buildings with broken windows** |  |  |
| None | 538 | (91.3) |
| A Few (1-3) | 48 | (8.1) |
| Some (4-6) | 2 | (0.3) |
| A Lot (7+) | 2 | (0.3) |

**Table A5.** Frequency distributions of select **signage** features, TRAIN Study, 2014.

| **Signage characteristics** | **Count** | **Percent** |
| --- | --- | --- |
| **Cultural or religious message or event** |  |  |
| None | 542 | (91.9) |
| A Few (1-3) | 44 | (7.5) |
| Some (4-6) | 2 | (0.3) |
| A Lot (7+) | 2 | (0.3) |
|  |  |  |
| **Political message or event** |  |  |
| None | 555 | (94.0) |
| A Few (1-3) | 34 | (5.8) |
| Some (4-6) | 0 | (0.0) |
| A Lot (7+) | 1 | (0.2) |
|  |  |  |
| **"Share the road" sign** |  |  |
| None | 567 | (96.1) |
| A Few (1-3) | 22 | (3.7) |
| Some (4-6) | 0 | (0.0) |
| A Lot (&gt;7) | 1 | (0.2) |
|  |  |  |
| **Other pedestrian or bicyclist friendly traffic sign** |  |  |
| None | 275 | (46.6) |
| A Few (1-3) | 292 | (49.5) |
| Some (4-6) | 21 | (3.6) |
| A Lot (7+) | 2 | (0.3) |
|  |  |  |
| **Security warning sign** |  |  |
| None | 500 | (84.7) |
| A Few (1-3) | 74 | (12.5) |
| Some (4-6) | 12 | (2.0) |
| A Lot (7+) | 4 | (0.7) |
|  |  |  |
| **No trespassing/beware of dog sign** |  |  |
| None | 394 | (66.7) |
| A Few (1-3) | 149 | (25.3) |
| Some (4-6) | 29 | (4.9) |
| A Lot (7+) | 18 | (3.1) |

**Table A6.** Frequency distributions of select **social environment** features, TRAIN Study, 2014.

| **Social environment** | **Count** | **Percent** |
| --- | --- | --- |
| **People visible in this segment** |  |  |
| None | 140 | (23.7) |
| A Few (1-3) | 261 | (44.2) |
| Some (4-6) | 77 | (13.1) |
| A Lot (7+) | 112 | (19.0) |
|  |  |  |
| **Children visible in this segment** |  |  |
| None | 547 | (92.7) |
| A Few (1-3) | 37 | (6.3) |
| Some (4-6) | 4 | (0.7) |
| A Lot (7+) | 2 | (0.3) |
|  |  |  |
| **Children engaging in active behaviors** |  |  |
| None | 559 | (94.8) |
| A Few (1-3) | 24 | (4.1) |
| Some (4-6) | 5 | (0.8) |
| A Lot (7+) | 2 | (0.3) |
|  |  |  |
| **Teenagers or adults visible in this segment (13-65 years old)** |  |  |
| None | 160 | (27.2) |
| A Few (1-3) | 274 | (46.4) |
| Some (4-6) | 71 | (12.0) |
| A Lot (7+) | 85 | (14.4) |
|  |  |  |
| **Teenagers or adults engaging in active behaviors** |  |  |
| None | 214 | (36.3) |
| A Few (1-3) | 241 | (40.8) |
| Some (4-6) | 62 | (10.5) |
| A Lot (7+) | 73 | (12.4) |
|  |  |  |
| **Older adults visible in this segment (older than 65)** |  |  |
| None | 513 | (87.0) |
| A Few (1-3) | 66 | (11.2) |
| Some (4-6) | 6 | (1.0) |
| A Lot (7+) | 5 | (0.8) |
|  |  |  |
| **Older adults engaging in active behaviors** |  |  |
| None | 518 | (87.8) |
| A Few (1-3) | 57 | (9.7) |
| Some (4-6) | 5 | (0.8) |
| A Lot (7+) | 10 | (1.7) |
|  |  |  |
| **Stray dogs or animals visible in this segment** |  |  |
| None | 522 | (88.5) |
| A Few (1-3) | 66 | (11.2) |
| Some (4-6) | 2 | (0.3) |
| A Lot (7+) | 0 | (0.0) |
