# Supplementary material for: Foot-based audit of streets adjacent to new light rail stations in Houston, Texas: measurement of health-related characteristics of the built environment for physical activity research
Source: BMC Public Health. 2019 Feb 28;19:238. doi: 10.1186/s12889-019-6560-4 (PMC6393971; doi:10.1186/s12889-019-6560-4)
Supplement: Supplementary file 2 — Calculating the Composite Index (C.I.) scores for the six domains that were represented in the environmental audit instrument. Houston TRAIN Study, 2014. This file contains several Tables that provide detailed information on how we calculated the composite index (C.I.) scores for all the six built environment domains that were represented in the environmental audit instrument. (DOCX 224 kb) [file 12889_2019_6560_MOESM2_ESM.docx]

**TITLE: Foot-based audit of streets adjacent to new light rail stations in Houston, Texas: Measurement of health-related characteristics of the built environment for physical activity research.**

**Supplemental Material (B): Calculating the Composite Index (C.I.) scores for the six domains that were represented in the environmental audit instrument. Houston TRAIN Study, 2014.**

**Table B1.** Calculating the Composite Index (C.I.) scores for the **entire study area** (590 segments). Houston TRAIN Study, 2014.

| **Descriptive statements** | | | Hardly ever | Rarely | Occasionally | Half the time | Frequently | Usually | Always | Composite Index^b^ | |
| --- | --- | --- | --- | --- | --- | --- | --- | --- | --- | --- | --- |
| **Percent ranges** | | | 0-10.9 | 11-30.9 | 31-45.9 | 46-55.9 | 56-70.9 | 71-90.9 | 91-100 |  |  |
| **Scores** | | | 1.00 | 2.00 | 3.00 | 4.00 | 5.00 | 6.00 | 7.00 |  |  |
|  | P/N^a^ | (%) |  |  |  |  |  |  |  |  | |
| **Land Use** |  |  |  |  |  |  |  |  |  |  |  |
| Land use integration | P | 27.1% | 0 | 2 | 0 | 0 | 0 | 0 | 0 | 1.25 | (P) |
| Office building | P | 10.3% | 1 | 0 | 0 | 0 | 0 | 0 | 0 |  |  |
| Other services – e.g. beautician and lawyer | P | 12.5% | 0 | 2 | 0 | 0 | 0 | 0 | 0 |  |  |
| Fast food restaurants | P | 8.6% | 1 | 0 | 0 | 0 | 0 | 0 | 0 |  |  |
| Strip mall | P | 5.9% | 1 | 0 | 0 | 0 | 0 | 0 | 0 |  |  |
| Transportation facility | P | 6.8% | 1 | 0 | 0 | 0 | 0 | 0 | 0 |  |  |
| Place of worship | P | 6.1% | 1 | 0 | 0 | 0 | 0 | 0 | 0 |  |  |
| Park | P | 4.2% | 1 | 0 | 0 | 0 | 0 | 0 | 0 |  |  |
| Warehouses, factories, or industrial buildings | N | 14.1% | 0 | 2 | 0 | 0 | 0 | 0 | 0 | 3.00 | (N) |
| Auto shop | N | 10.2% | 1 | 0 | 0 | 0 | 0 | 0 | 0 |  |  |
| Parking lot or parking garage | N | 50.3% | 0 | 0 | 0 | 4 | 0 | 0 | 0 |  |  |
| Driveway (either residential or non-residential) | N | 89.5% | 0 | 0 | 0 | 0 | 0 | 6 | 0 |  |  |
| Abandoned building or vacant lot | N | 44.2% | 0 | 0 | 3 | 0 | 0 | 0 | 0 |  |  |
| Major transportation development (e.g. bridge, tunnel) | N | 14.2% | 0 | 2 | 0 | 0 | 0 | 0 | 0 |  |  |
| **Transportation** |  |  |  |  |  |  |  |  |  |  |  |
| Any alternative transportation modes | P | 76.6% | 0 | 0 | 0 | 0 | 0 | 6 | 0 | 4.06 | (P) |
| Any sidewalk facility | P | 76.9% | 0 | 0 | 0 | 0 | 0 | 6 | 0 |  |  |
| Sidewalk continuity; at least 1 side of street (both ends)^c^ | P | 67.0% | 0 | 0 | 0 | 0 | 5 | 0 | 0 |  |  |
| Curvilinear curbs; at least 1 side of street (both ends)**^c^** | P | 60.1% | 0 | 0 | 0 | 0 | 5 | 0 | 0 |  |  |
| Sidewalk coverage on left side of segment (>75%)^c^ | P | 74.9% | 0 | 0 | 0 | 0 | 0 | 6 | 0 |  |  |
| Sidewalk coverage on right side of segment (>75%)^c^ | P | 70.9% | 0 | 0 | 0 | 0 | 5 | 0 | 0 |  |  |
| Bike lane or marked shoulder (for bikes) | P | 2.7% | 1 | 0 | 0 | 0 | 0 | 0 | 0 |  |  |
| Bike racks present | P | 2.5% | 1 | 0 | 0 | 0 | 0 | 0 | 0 |  |  |
| Bus/transit stop present | P | 20.5% | 0 | 2 | 0 | 0 | 0 | 0 | 0 |  |  |
| Bus stop; covered shelter with/out bench^c^ | P | 60.3% | 0 | 0 | 0 | 0 | 5 | 0 | 0 |  |  |
| Non-concrete multi-use trails or paths | P | 4.4% | 1 | 0 | 0 | 0 | 0 | 0 | 0 |  |  |
| Posted speed limit | P | 7.1% | 1 | 0 | 0 | 0 | 0 | 0 | 0 |  |  |
| On-street parking | P | 36.8% | 0 | 0 | 3 | 0 | 0 | 0 | 0 |  |  |
| >3 directions at intersections | P | 88.6% | 0 | 0 | 0 | 0 | 0 | 6 | 0 |  |  |
| Road design to reduce car volume/speed | P | 17.8% | 0 | 2 | 0 | 0 | 0 | 0 | 0 |  |  |
| Traffic calming devices to reduce car volume/speed | P | 80.5% | 0 | 0 | 0 | 0 | 0 | 6 | 0 |  |  |
| Crossing aids for pedestrian / bicyclist | P | 66.1% | 0 | 0 | 0 | 0 | 5 | 0 | 0 |  |  |
| Street lighting for sidewalk, street shoulders, etc. | P | 96.1% | 0 | 0 | 0 | 0 | 0 | 0 | 7 |  |  |
| Sidewalk has heaves, cracks, broken sections, etc.^c^ | N | 34.8% | 0 | 0 | 3 | 0 | 0 | 0 | 0 | 2.50 | (N) |
| Sidewalk blocked by obstacles^c^ | N | 28.2% | 0 | 2 | 0 | 0 | 0 | 0 | 0 |  |  |
| **Facilities** |  |  |  |  |  |  |  |  |  |  |  |
| Public / recreational facilities | P | 8.5% | 1 | 0 | 0 | 0 | 0 | 0 | 0 | 1.00 | (P) |
| Public / recreational equipment | P | 6.1% | 1 | 0 | 0 | 0 | 0 | 0 | 0 |  |  |
| Playground equipment | P | 3.9% | 1 | 0 | 0 | 0 | 0 | 0 | 0 |  |  |
| **Aesthetics** |  |  |  |  |  |  |  |  |  |  |  |
| Attractive features (e.g., architecture, vegetation) | P | 62.0% | 0 | 0 | 0 | 0 | 5 | 0 | 0 | 5.50 | (P) |
| Comfort features (shade, trees, benches, etc.) | P | 82.9% | 0 | 0 | 0 | 0 | 0 | 6 | 0 |  |  |
| Air pollution | N | 9.2% | 1 | 0 | 0 | 0 | 0 | 0 | 0 | 4.17 | (N) |
| Noise pollution | N | 38.3% | 0 | 0 | 3 | 0 | 0 | 0 | 0 |  |  |
| Physical disorder (general) | N | 79.7% | 0 | 0 | 0 | 0 | 0 | 6 | 0 |  |  |
| Whole or broken beer or liquor bottles or can | N | 42.9% | 0 | 0 | 3 | 0 | 0 | 0 | 0 |  |  |
| Cigarette, cigar butts or discarded cigarette packages | N | 71.0% | 0 | 0 | 0 | 0 | 0 | 6 | 0 |  |  |
| Garbage, litter, or broken glass | N | 73.7% | 0 | 0 | 0 | 0 | 0 | 6 | 0 |  |  |
| **Signage** |  |  |  |  |  |  |  |  |  |  |  |
| Pedestrian or bicyclist friendly traffic signs | P | 53.4% | 0 | 0 | 0 | 4 | 0 | 0 | 0 | 1.60 | (P) |
| Share the road sign | P | 3.9% | 1 | 0 | 0 | 0 | 0 | 0 | 0 |  |  |
| Religious message | P | 8.1% | 1 | 0 | 0 | 0 | 0 | 0 | 0 |  |  |
| Political message | P | 5.9% | 1 | 0 | 0 | 0 | 0 | 0 | 0 |  |  |
| Fast food billboard | P | 6.6% | 1 | 0 | 0 | 0 | 0 | 0 | 0 |  |  |
| No trespassing / beware of dogs | N | 33.2% | 0 | 0 | 3 | 0 | 0 | 0 | 0 | 2.50 | (N) |
| Security warning | N | 15.3% | 0 | 2 | 0 | 0 | 0 | 0 | 0 |  |  |
| **Social Environment** |  |  |  |  |  |  |  |  |  |  |  |
| Children present in the street | P | 7.3% | 1 | 0 | 0 | 0 | 0 | 0 | 0 | 2.83 | (P) |
| Children engaged in active behavior | P | 5.3% | 1 | 0 | 0 | 0 | 0 | 0 | 0 |  |  |
| Teenagers/adults present in the street | P | 72.9% | 0 | 0 | 0 | 0 | 0 | 6 | 0 |  |  |
| Teenagers/adults engaged in active behavior | P | 63.9% | 0 | 0 | 0 | 0 | 5 | 0 | 0 |  |  |
| Older adults (>65 years) present in the street | P | 13.1% | 0 | 2 | 0 | 0 | 0 | 0 | 0 |  |  |
| Older adults (>65 years) engaged in active behavior | P | 12.2% | 0 | 2 | 0 | 0 | 0 | 0 | 0 |  |  |
| Stray dogs or animals (not squirrels or rabbits) | N | 11.5% | 0 | 2 | 0 | 0 | 0 | 0 | 0 | 2.00 | (N) |

Abbreviations: TRAIN, Travel Related Activity in Neighborhoods.

a. Observed features were arranged into two classes: positive (P) and negative (N). This classification was based on the expected direction of the relationship between each feature and physical activity behavior, as evidenced by findings in available literature.

b. C.I. scores were calculated separately for each class (positive vs. negative) in each domain. To calculate a C.I. score, we first dichotomized the observed categories for selected features, such that features with multiple categories were collapsed into “any observation” versus “no observation. Thereafter, we calculated the percentage of all the audited segments in the entire study area with “any observation” of these selected features. We then converted the calculated percentage to seven descriptive statements that ranged from “hardly ever” to “always”. For example, a feature that was observed in 0-10.9 percent of the audited segments was labeled "hardly ever," and so on. The seven descriptive statements were thereafter assigned scores, ranging from 1.00 for "hardly ever" (0%-10.9%) to 7.00 for "always" (91%-100%). We then calculated the average score for that class and maintained the min=1.00, max=7.00 average score (i.e., the C.I. score). Where positive relationship with physical activity is expected, a high C.I. score is indicative of prevalence of physical activity friendly features, whereas, for negative relationships, a high score indicates prevalence non-physical activity friendly features.

c. Data shown (percentages) are based on a subset of the audited 590 segments that are applicable to these particular audit questions. For example. Sidewalk continuity only applies to segments with sidewalks.

**Table B2.** Calculating the Composite Index (C.I.) scores for the **Northside/Northline** super neighborhood (28 segments). Houston TRAIN Study, 2014.

| **Descriptive statements** | | | Hardly ever | Rarely | Occasionally | Half the time | Frequently | Usually | Always | Composite Index^b^ | |
| --- | --- | --- | --- | --- | --- | --- | --- | --- | --- | --- | --- |
| **Percent ranges** | | | 0-10.9 | 11-30.9 | 31-45.9 | 46-55.9 | 56-70.9 | 71-90.9 | 91-100 |  |  |
| **Scores** | | | 1.00 | 2.00 | 3.00 | 4.00 | 5.00 | 6.00 | 7.00 |  |  |
|  | P/N^a^ | (%) |  |  |  |  |  |  |  |  | |
| **Land Use** |  |  |  |  |  |  |  |  |  |  |  |
| Land use integration | P | 50.0% | 0 | 0 | 0 | 4 | 0 | 0 | 0 | 2.00 | (P) |
| Office building | P | 7.1% | 1 | 0 | 0 | 0 | 0 | 0 | 0 |  |  |
| Other services – e.g. beautician and lawyer | P | 32.1% | 0 | 0 | 3 | 0 | 0 | 0 | 0 |  |  |
| Fast food restaurants | P | 21.4% | 0 | 2 | 0 | 0 | 0 | 0 | 0 |  |  |
| Strip mall | P | 17.9% | 0 | 2 | 0 | 0 | 0 | 0 | 0 |  |  |
| Transportation facility | P | 21.4% | 0 | 2 | 0 | 0 | 0 | 0 | 0 |  |  |
| Place of worship | P | 7.1% | 1 | 0 | 0 | 0 | 0 | 0 | 0 |  |  |
| Park | P | 10.7% | 1 | 0 | 0 | 0 | 0 | 0 | 0 |  |  |
| Warehouses, factories, or industrial buildings | N | 21.4% | 0 | 2 | 0 | 0 | 0 | 0 | 0 | 3.83 | (N) |
| Auto shop | N | 35.7% | 0 | 0 | 3 | 0 | 0 | 0 | 0 |  |  |
| Parking lot or parking garage | N | 71.4% | 0 | 0 | 0 | 0 | 0 | 6 | 0 |  |  |
| Driveway (either residential or non-residential) | N | 92.9% | 0 | 0 | 0 | 0 | 0 | 0 | 7 |  |  |
| Abandoned building or vacant lot | N | 50.0% | 0 | 0 | 0 | 4 | 0 | 0 | 0 |  |  |
| Major transportation development (e.g. bridge, tunnel) | N | 10.7% | 1 | 0 | 0 | 0 | 0 | 0 | 0 |  |  |
| **Transportation** |  |  |  |  |  |  |  |  |  |  |  |
| Any alternative transportation modes | P | 82.1% | 0 | 0 | 0 | 0 | 0 | 6 | 0 | 3.72 | (P) |
| Any sidewalk facility | P | 89.3% | 0 | 0 | 0 | 0 | 0 | 6 | 0 |  |  |
| Sidewalk continuity; at least 1 side of street (both ends)^c^ | P | 48.0% | 0 | 0 | 0 | 4 | 0 | 0 | 0 |  |  |
| Curvilinear curbs; at least 1 side of street (both ends)**^c^** | P | 40.0% | 0 | 0 | 3 | 0 | 0 | 0 | 0 |  |  |
| Sidewalk coverage on left side of segment (>75%)^c^ | P | 56.0% | 0 | 0 | 0 | 0 | 5 | 0 | 0 |  |  |
| Sidewalk coverage on right side of segment (>75%)^c^ | P | 28.0% | 0 | 2 | 0 | 0 | 0 | 0 | 0 |  |  |
| Bike lane or marked shoulder (for bikes) | P | 3.6% | 1 | 0 | 0 | 0 | 0 | 0 | 0 |  |  |
| Bike racks present | P | 0.0% | 1 | 0 | 0 | 0 | 0 | 0 | 0 |  |  |
| Bus/transit stop present | P | 35.7% | 0 | 0 | 3 | 0 | 0 | 0 | 0 |  |  |
| Bus stop; covered shelter with/out bench^c^ | P | 70.0% | 0 | 0 | 0 | 0 | 5 | 0 | 0 |  |  |
| Non-concrete multi-use trails or paths | P | 14.3% | 0 | 2 | 0 | 0 | 0 | 0 | 0 |  |  |
| Posted speed limit | P | 10.7% | 1 | 0 | 0 | 0 | 0 | 0 | 0 |  |  |
| On-street parking | P | 21.4% | 0 | 2 | 0 | 0 | 0 | 0 | 0 |  |  |
| >3 directions at intersections | P | 78.6% | 0 | 0 | 0 | 0 | 0 | 6 | 0 |  |  |
| Road design to reduce car volume/speed | P | 7.1% | 1 | 0 | 0 | 0 | 0 | 0 | 0 |  |  |
| Traffic calming devices to reduce car volume/speed | P | 82.1% | 0 | 0 | 0 | 0 | 0 | 6 | 0 |  |  |
| Crossing aids for pedestrian / bicyclist | P | 75.0% | 0 | 0 | 0 | 0 | 0 | 6 | 0 |  |  |
| Street lighting for sidewalk, street shoulders, etc. | P | 96.4% | 0 | 0 | 0 | 0 | 0 | 0 | 7 |  |  |
| Sidewalk has heaves, cracks, broken sections, etc.^c^ | N | 4.0% | 1 | 0 | 0 | 0 | 0 | 0 | 0 | 1.00 | (N) |
| Sidewalk blocked by obstacles^c^ | N | 0.0% | 1 | 0 | 0 | 0 | 0 | 0 | 0 |  |  |
| **Facilities** |  |  |  |  |  |  |  |  |  |  |  |
| Public / recreational facilities | P | 25.0% | 0 | 2 | 0 | 0 | 0 | 0 | 0 | 1.67 | (P) |
| Public / recreational equipment | P | 21.4% | 0 | 2 | 0 | 0 | 0 | 0 | 0 |  |  |
| Playground equipment | P | 10.7% | 1 | 0 | 0 | 0 | 0 | 0 | 0 |  |  |
| **Aesthetics** |  |  |  |  |  |  |  |  |  |  |  |
| Attractive features (e.g., architecture, vegetation) | P | 60.7% | 0 | 0 | 0 | 0 | 5 | 0 | 0 | 5.50 | (P) |
| Comfort features (shade, trees, benches, etc.) | P | 85.7% | 0 | 0 | 0 | 0 | 0 | 6 | 0 |  |  |
| Air pollution | N | 25.0% | 0 | 2 | 0 | 0 | 0 | 0 | 0 | 4.50 | (N) |
| Noise pollution | N | 32.1% | 0 | 0 | 3 | 0 | 0 | 0 | 0 |  |  |
| Physical disorder (general) | N | 89.3% | 0 | 0 | 0 | 0 | 0 | 6 | 0 |  |  |
| Whole or broken beer or liquor bottles or can | N | 53.6% | 0 | 0 | 0 | 4 | 0 | 0 | 0 |  |  |
| Cigarette, cigar butts or discarded cigarette packages | N | 85.7% | 0 | 0 | 0 | 0 | 0 | 6 | 0 |  |  |
| Garbage, litter, or broken glass | N | 82.1% | 0 | 0 | 0 | 0 | 0 | 6 | 0 |  |  |
| **Signage** |  |  |  |  |  |  |  |  |  |  |  |
| Pedestrian or bicyclist friendly traffic signs | P | 46.4% | 0 | 0 | 0 | 4 | 0 | 0 | 0 | 1.80 | (P) |
| Share the road sign | P | 7.1% | 1 | 0 | 0 | 0 | 0 | 0 | 0 |  |  |
| Religious message | P | 7.1% | 1 | 0 | 0 | 0 | 0 | 0 | 0 |  |  |
| Political message | P | 3.6% | 1 | 0 | 0 | 0 | 0 | 0 | 0 |  |  |
| Fast food billboard | P | 25.0% | 0 | 2 | 0 | 0 | 0 | 0 | 0 |  |  |
| No trespassing / beware of dogs | N | 57.1% | 0 | 0 | 0 | 0 | 5 | 0 | 0 | 3.50 | (N) |
| Security warning | N | 17.9% | 0 | 2 | 0 | 0 | 0 | 0 | 0 |  |  |
| **Social Environment** |  |  |  |  |  |  |  |  |  |  |  |
| Children present in the street | P | 7.1% | 1 | 0 | 0 | 0 | 0 | 0 | 0 | 3.17 | (P) |
| Children engaged in active behavior | P | 3.6% | 1 | 0 | 0 | 0 | 0 | 0 | 0 |  |  |
| Teenagers/adults present in the street | P | 92.9% | 0 | 0 | 0 | 0 | 0 | 0 | 7 |  |  |
| Teenagers/adults engaged in active behavior | P | 78.6% | 0 | 0 | 0 | 0 | 0 | 6 | 0 |  |  |
| Older adults (>65 years) present in the street | P | 14.3% | 0 | 2 | 0 | 0 | 0 | 0 | 0 |  |  |
| Older adults (>65 years) engaged in active behavior | P | 14.3% | 0 | 2 | 0 | 0 | 0 | 0 | 0 |  |  |
| Stray dogs or animals (not squirrels or rabbits) | N | 28.6% | 0 | 2 | 0 | 0 | 0 | 0 | 0 | 2.00 | (N) |

Abbreviations: TRAIN, Travel Related Activity in Neighborhoods.

a. Observed features were arranged into two classes: positive (P) and negative (N). This classification was based on the expected direction of the relationship between each feature and physical activity behavior, as evidenced by findings in available literature.

b. C.I. scores were calculated separately for each class (positive vs. negative) in each domain. To calculate a C.I. score, we first dichotomized the observed categories for selected features, such that features with multiple categories were collapsed into “any observation” versus “no observation. Thereafter, we calculated the percentage of all the audited segments in the entire study area with “any observation” of these selected features. We then converted the calculated percentage to seven descriptive statements that ranged from “hardly ever” to “always”. For example, a feature that was observed in 0-10.9 percent of the audited segments was labeled "hardly ever," and so on. The seven descriptive statements were thereafter assigned scores, ranging from 1.00 for "hardly ever" (0%-10.9%) to 7.00 for "always" (91%-100%). We then calculated the average score for that class and maintained the min=1.00, max=7.00 average score (i.e., the C.I. score). Where positive relationship with physical activity is expected, a high C.I. score is indicative of prevalence of physical activity friendly features, whereas, for negative relationships, a high score indicates prevalence non-physical activity friendly features.

c. Data shown (percentages) are based on a subset of the audited 590 segments that are applicable to these particular audit questions. For example. Sidewalk continuity only applies to segments with sidewalks.

**Table B3.** Calculating the Composite Index (C.I.) scores for the **Northside Village** super neighborhood (214 segments). Houston TRAIN Study, 2014.

| **Descriptive statements** | | | Hardly ever | Rarely | Occasionally | Half the time | Frequently | Usually | Always | Composite Index^b^ | |
| --- | --- | --- | --- | --- | --- | --- | --- | --- | --- | --- | --- |
| **Percent ranges** | | | 0-10.9 | 11-30.9 | 31-45.9 | 46-55.9 | 56-70.9 | 71-90.9 | 91-100 |  |  |
| **Scores** | | | 1.00 | 2.00 | 3.00 | 4.00 | 5.00 | 6.00 | 7.00 |  |  |
|  | P/N^a^ | (%) |  |  |  |  |  |  |  |  | |
| **Land Use** |  |  |  |  |  |  |  |  |  |  |  |
| Land use integration | P | 36.0% | 0 | 0 | 3 | 0 | 0 | 0 | 0 | 1.25 | (P) |
| Office building | P | 3.7% | 1 | 0 | 0 | 0 | 0 | 0 | 0 |  |  |
| Other services – e.g. beautician and lawyer | P | 10.7% | 1 | 0 | 0 | 0 | 0 | 0 | 0 |  |  |
| Fast food restaurants | P | 6.1% | 1 | 0 | 0 | 0 | 0 | 0 | 0 |  |  |
| Strip mall | P | 5.6% | 1 | 0 | 0 | 0 | 0 | 0 | 0 |  |  |
| Transportation facility | P | 3.7% | 1 | 0 | 0 | 0 | 0 | 0 | 0 |  |  |
| Place of worship | P | 7.5% | 1 | 0 | 0 | 0 | 0 | 0 | 0 |  |  |
| Park | P | 3.3% | 1 | 0 | 0 | 0 | 0 | 0 | 0 |  |  |
| Warehouses, factories, or industrial buildings | N | 12.1% | 0 | 2 | 0 | 0 | 0 | 0 | 0 | 3.17 | (N) |
| Auto shop | N | 9.3% | 1 | 0 | 0 | 0 | 0 | 0 | 0 |  |  |
| Parking lot or parking garage | N | 42.1% | 0 | 0 | 3 | 0 | 0 | 0 | 0 |  |  |
| Driveway (either residential or non-residential) | N | 91.6% | 0 | 0 | 0 | 0 | 0 | 0 | 7 |  |  |
| Abandoned building or vacant lot | N | 48.6% | 0 | 0 | 0 | 4 | 0 | 0 | 0 |  |  |
| Major transportation development (e.g. bridge, tunnel) | N | 14.0% | 0 | 2 | 0 | 0 | 0 | 0 | 0 |  |  |
| **Transportation** |  |  |  |  |  |  |  |  |  |  |  |
| Any alternative transportation modes | P | 64.0% | 0 | 0 | 0 | 0 | 5 | 0 | 0 | 3.72 | (P) |
| Any sidewalk facility | P | 63.1% | 0 | 0 | 0 | 0 | 5 | 0 | 0 |  |  |
| Sidewalk continuity; at least 1 side of street (both ends)^c^ | P | 63.7% | 0 | 0 | 0 | 0 | 5 | 0 | 0 |  |  |
| Curvilinear curbs; at least 1 side of street (both ends)**^c^** | P | 39.3% | 0 | 0 | 3 | 0 | 0 | 0 | 0 |  |  |
| Sidewalk coverage on left side of segment (>75%)^c^ | P | 65.9% | 0 | 0 | 0 | 0 | 5 | 0 | 0 |  |  |
| Sidewalk coverage on right side of segment (>75%)^c^ | P | 63.7% | 0 | 0 | 0 | 0 | 5 | 0 | 0 |  |  |
| Bike lane or marked shoulder (for bikes) | P | 2.8% | 1 | 0 | 0 | 0 | 0 | 0 | 0 |  |  |
| Bike racks present | P | 0.5% | 1 | 0 | 0 | 0 | 0 | 0 | 0 |  |  |
| Bus/transit stop present | P | 11.7% | 0 | 2 | 0 | 0 | 0 | 0 | 0 |  |  |
| Bus stop; covered shelter with/out bench^c^ | P | 68.0% | 0 | 0 | 0 | 0 | 5 | 0 | 0 |  |  |
| Non-concrete multi-use trails or paths | P | 1.4% | 1 | 0 | 0 | 0 | 0 | 0 | 0 |  |  |
| Posted speed limit | P | 7.9% | 1 | 0 | 0 | 0 | 0 | 0 | 0 |  |  |
| On-street parking | P | 38.8% | 0 | 0 | 3 | 0 | 0 | 0 | 0 |  |  |
| >3 directions at intersections | P | 87.4% | 0 | 0 | 0 | 0 | 0 | 6 | 0 |  |  |
| Road design to reduce car volume/speed | P | 10.3% | 1 | 0 | 0 | 0 | 0 | 0 | 0 |  |  |
| Traffic calming devices to reduce car volume/speed | P | 73.8% | 0 | 0 | 0 | 0 | 0 | 6 | 0 |  |  |
| Crossing aids for pedestrian / bicyclist | P | 60.3% | 0 | 0 | 0 | 0 | 5 | 0 | 0 |  |  |
| Street lighting for sidewalk, street shoulders, etc. | P | 95.3% | 0 | 0 | 0 | 0 | 0 | 0 | 7 |  |  |
| Sidewalk has heaves, cracks, broken sections, etc.^c^ | N | 39.3% | 0 | 0 | 3 | 0 | 0 | 0 | 0 | 2.50 | (N) |
| Sidewalk blocked by obstacles^c^ | N | 23.7% | 0 | 2 | 0 | 0 | 0 | 0 | 0 |  |  |
| **Facilities** |  |  |  |  |  |  |  |  |  |  |  |
| Public / recreational facilities | P | 5.1% | 1 | 0 | 0 | 0 | 0 | 0 | 0 | 1.00 | (P) |
| Public / recreational equipment | P | 6.5% | 1 | 0 | 0 | 0 | 0 | 0 | 0 |  |  |
| Playground equipment | P | 6.1% | 1 | 0 | 0 | 0 | 0 | 0 | 0 |  |  |
| **Aesthetics** |  |  |  |  |  |  |  |  |  |  |  |
| Attractive features (e.g., architecture, vegetation) | P | 52.3% | 0 | 0 | 0 | 4 | 0 | 0 | 0 | 5.00 | (P) |
| Comfort features (shade, trees, benches, etc.) | P | 84.1% | 0 | 0 | 0 | 0 | 0 | 6 | 0 |  |  |
| Air pollution | N | 7.5% | 1 | 0 | 0 | 0 | 0 | 0 | 0 | 3.83 | (N) |
| Noise pollution | N | 30.4% | 0 | 2 | 0 | 0 | 0 | 0 | 0 |  |  |
| Physical disorder (general) | N | 78.0% | 0 | 0 | 0 | 0 | 0 | 6 | 0 |  |  |
| Whole or broken beer or liquor bottles or can | N | 39.3% | 0 | 0 | 3 | 0 | 0 | 0 | 0 |  |  |
| Cigarette, cigar butts or discarded cigarette packages | N | 63.1% | 0 | 0 | 0 | 0 | 5 | 0 | 0 |  |  |
| Garbage, litter, or broken glass | N | 79.4% | 0 | 0 | 0 | 0 | 0 | 6 | 0 |  |  |
| **Signage** |  |  |  |  |  |  |  |  |  |  |  |
| Pedestrian or bicyclist friendly traffic signs | P | 48.6% | 0 | 0 | 0 | 4 | 0 | 0 | 0 | 1.60 | (P) |
| Share the road sign | P | 4.2% | 1 | 0 | 0 | 0 | 0 | 0 | 0 |  |  |
| Religious message | P | 5.6% | 1 | 0 | 0 | 0 | 0 | 0 | 0 |  |  |
| Political message | P | 6.1% | 1 | 0 | 0 | 0 | 0 | 0 | 0 |  |  |
| Fast food billboard | P | 2.8% | 1 | 0 | 0 | 0 | 0 | 0 | 0 |  |  |
| No trespassing / beware of dogs | N | 37.4% | 0 | 0 | 3 | 0 | 0 | 0 | 0 | 2.50 | (N) |
| Security warning | N | 15.4% | 0 | 2 | 0 | 0 | 0 | 0 | 0 |  |  |
| **Social Environment** |  |  |  |  |  |  |  |  |  |  |  |
| Children present in the street | P | 7.0% | 1 | 0 | 0 | 0 | 0 | 0 | 0 | 2.67 | (P) |
| Children engaged in active behavior | P | 3.7% | 1 | 0 | 0 | 0 | 0 | 0 | 0 |  |  |
| Teenagers/adults present in the street | P | 67.8% | 0 | 0 | 0 | 0 | 5 | 0 | 0 |  |  |
| Teenagers/adults engaged in active behavior | P | 57.9% | 0 | 0 | 0 | 0 | 5 | 0 | 0 |  |  |
| Older adults (>65 years) present in the street | P | 11.7% | 0 | 2 | 0 | 0 | 0 | 0 | 0 |  |  |
| Older adults (>65 years) engaged in active behavior | P | 11.2% | 0 | 2 | 0 | 0 | 0 | 0 | 0 |  |  |
| Stray dogs or animals (not squirrels or rabbits) | N | 13.1% | 0 | 2 | 0 | 0 | 0 | 0 | 0 | 2.00 | (N) |

Abbreviations: TRAIN, Travel Related Activity in Neighborhoods.

a. Observed features were arranged into two classes: positive (P) and negative (N). This classification was based on the expected direction of the relationship between each feature and physical activity behavior, as evidenced by findings in available literature.

b. C.I. scores were calculated separately for each class (positive vs. negative) in each domain. To calculate a C.I. score, we first dichotomized the observed categories for selected features, such that features with multiple categories were collapsed into “any observation” versus “no observation. Thereafter, we calculated the percentage of all the audited segments in the entire study area with “any observation” of these selected features. We then converted the calculated percentage to seven descriptive statements that ranged from “hardly ever” to “always”. For example, a feature that was observed in 0-10.9 percent of the audited segments was labeled "hardly ever," and so on. The seven descriptive statements were thereafter assigned scores, ranging from 1.00 for "hardly ever" (0%-10.9%) to 7.00 for "always" (91%-100%). We then calculated the average score for that class and maintained the min=1.00, max=7.00 average score (i.e., the C.I. score). Where positive relationship with physical activity is expected, a high C.I. score is indicative of prevalence of physical activity friendly features, whereas, for negative relationships, a high score indicates prevalence non-physical activity friendly features.

c. Data shown (percentages) are based on a subset of the audited 590 segments that are applicable to these particular audit questions. For example. Sidewalk continuity only applies to segments with sidewalks.

**Table B5.** Calculating the Composite Index (C.I.) scores for the **Downtown** super neighborhood (118 segments). Houston TRAIN Study, 2014.

| **Descriptive statements** | | | Hardly ever | Rarely | Occasionally | Half the time | Frequently | Usually | Always | Composite Index^b^ | |
| --- | --- | --- | --- | --- | --- | --- | --- | --- | --- | --- | --- |
| **Percent ranges** | | | 0-10.9 | 11-30.9 | 31-45.9 | 46-55.9 | 56-70.9 | 71-90.9 | 91-100 |  |  |
| **Scores** | | | 1.00 | 2.00 | 3.00 | 4.00 | 5.00 | 6.00 | 7.00 |  |  |
|  | P/N^a^ | (%) |  |  |  |  |  |  |  |  | |
| **Land Use** |  |  |  |  |  |  |  |  |  |  |  |
| Land use integration | P | 7.6% | 1 | 0 | 0 | 0 | 0 | 0 | 0 | 1.25 | (P) |
| Office building | P | 32.2% | 0 | 0 | 3 | 0 | 0 | 0 | 0 |  |  |
| Other services – e.g. beautician and lawyer | P | 9.3% | 1 | 0 | 0 | 0 | 0 | 0 | 0 |  |  |
| Fast food restaurants | P | 8.5% | 1 | 0 | 0 | 0 | 0 | 0 | 0 |  |  |
| Strip mall | P | 1.7% | 1 | 0 | 0 | 0 | 0 | 0 | 0 |  |  |
| Transportation facility | P | 7.6% | 1 | 0 | 0 | 0 | 0 | 0 | 0 |  |  |
| Place of worship | P | 4.2% | 1 | 0 | 0 | 0 | 0 | 0 | 0 |  |  |
| Park | P | 5.9% | 1 | 0 | 0 | 0 | 0 | 0 | 0 |  |  |
| Warehouses, factories, or industrial buildings | N | 13.6% | 0 | 2 | 0 | 0 | 0 | 0 | 0 | 3.00 | (N) |
| Auto shop | N | 0.0% | 1 | 0 | 0 | 0 | 0 | 0 | 0 |  |  |
| Parking lot or parking garage | N | 68.6% | 0 | 0 | 0 | 0 | 5 | 0 | 0 |  |  |
| Driveway (either residential or non-residential) | N | 76.3% | 0 | 0 | 0 | 0 | 0 | 6 | 0 |  |  |
| Abandoned building or vacant lot | N | 18.6% | 0 | 2 | 0 | 0 | 0 | 0 | 0 |  |  |
| Major transportation development (e.g. bridge, tunnel) | N | 19.5% | 0 | 2 | 0 | 0 | 0 | 0 | 0 |  |  |
| **Transportation** |  |  |  |  |  |  |  |  |  |  |  |
| Any alternative transportation modes | P | 100.0% | 0 | 0 | 0 | 0 | 0 | 0 | 7 | 4.67 | (P) |
| Any sidewalk facility | P | 99.2% | 0 | 0 | 0 | 0 | 0 | 0 | 7 |  |  |
| Sidewalk continuity; at least 1 side of street (both ends)^c^ | P | 85.5% | 0 | 0 | 0 | 0 | 0 | 6 | 0 |  |  |
| Curvilinear curbs; at least 1 side of street (both ends)**^c^** | P | 92.3% | 0 | 0 | 0 | 0 | 0 | 0 | 7 |  |  |
| Sidewalk coverage on left side of segment (>75%)^c^ | P | 89.7% | 0 | 0 | 0 | 0 | 0 | 6 | 0 |  |  |
| Sidewalk coverage on right side of segment (>75%)^c^ | P | 86.3% | 0 | 0 | 0 | 0 | 0 | 6 | 0 |  |  |
| Bike lane or marked shoulder (for bikes) | P | 0.8% | 1 | 0 | 0 | 0 | 0 | 0 | 0 |  |  |
| Bike racks present | P | 9.3% | 1 | 0 | 0 | 0 | 0 | 0 | 0 |  |  |
| Bus/transit stop present | P | 33.1% | 0 | 0 | 3 | 0 | 0 | 0 | 0 |  |  |
| Bus stop; covered shelter with/out bench^c^ | P | 59.0% | 0 | 0 | 0 | 0 | 5 | 0 | 0 |  |  |
| Non-concrete multi-use trails or paths | P | 3.4% | 1 | 0 | 0 | 0 | 0 | 0 | 0 |  |  |
| Posted speed limit | P | 0.0% | 1 | 0 | 0 | 0 | 0 | 0 | 0 |  |  |
| On-street parking | P | 39.8% | 0 | 0 | 3 | 0 | 0 | 0 | 0 |  |  |
| >3 directions at intersections | P | 98.3% | 0 | 0 | 0 | 0 | 0 | 0 | 7 |  |  |
| Road design to reduce car volume/speed | P | 15.3% | 0 | 2 | 0 | 0 | 0 | 0 | 0 |  |  |
| Traffic calming devices to reduce car volume/speed | P | 94.9% | 0 | 0 | 0 | 0 | 0 | 0 | 7 |  |  |
| Crossing aids for pedestrian / bicyclist | P | 91.5% | 0 | 0 | 0 | 0 | 0 | 0 | 7 |  |  |
| Street lighting for sidewalk, street shoulders, etc. | P | 96.6% | 0 | 0 | 0 | 0 | 0 | 0 | 7 |  |  |
| Sidewalk has heaves, cracks, broken sections, etc.^c^ | N | 14.5% | 0 | 2 | 0 | 0 | 0 | 0 | 0 | 2.00 | (N) |
| Sidewalk blocked by obstacles^c^ | N | 21.4% | 0 | 2 | 0 | 0 | 0 | 0 | 0 |  |  |
| **Facilities** |  |  |  |  |  |  |  |  |  |  |  |
| Public / recreational facilities | P | 13.6% | 0 | 2 | 0 | 0 | 0 | 0 | 0 | 1.33 | (P) |
| Public / recreational equipment | P | 3.4% | 1 | 0 | 0 | 0 | 0 | 0 | 0 |  |  |
| Playground equipment | P | 0.0% | 1 | 0 | 0 | 0 | 0 | 0 | 0 |  |  |
| **Aesthetics** |  |  |  |  |  |  |  |  |  |  |  |
| Attractive features (e.g., architecture, vegetation) | P | 87.3% | 0 | 0 | 0 | 0 | 0 | 6 | 0 | 6.00 | (P) |
| Comfort features (shade, trees, benches, etc.) | P | 83.1% | 0 | 0 | 0 | 0 | 0 | 6 | 0 |  |  |
| Air pollution | N | 11.9% | 0 | 2 | 0 | 0 | 0 | 0 | 0 | 4.50 | (N) |
| Noise pollution | N | 79.7% | 0 | 0 | 0 | 0 | 0 | 6 | 0 |  |  |
| Physical disorder (general) | N | 74.6% | 0 | 0 | 0 | 0 | 0 | 6 | 0 |  |  |
| Whole or broken beer or liquor bottles or can | N | 27.1% | 0 | 2 | 0 | 0 | 0 | 0 | 0 |  |  |
| Cigarette, cigar butts or discarded cigarette packages | N | 81.4% | 0 | 0 | 0 | 0 | 0 | 6 | 0 |  |  |
| Garbage, litter, or broken glass | N | 62.7% | 0 | 0 | 0 | 0 | 5 | 0 | 0 |  |  |
| **Signage** |  |  |  |  |  |  |  |  |  |  |  |
| Pedestrian or bicyclist friendly traffic signs | P | 70.3% | 0 | 0 | 0 | 0 | 5 | 0 | 0 | 2.00 | (P) |
| Share the road sign | P | 5.9% | 1 | 0 | 0 | 0 | 0 | 0 | 0 |  |  |
| Religious message | P | 16.1% | 0 | 2 | 0 | 0 | 0 | 0 | 0 |  |  |
| Political message | P | 6.8% | 1 | 0 | 0 | 0 | 0 | 0 | 0 |  |  |
| Fast food billboard | P | 6.8% | 1 | 0 | 0 | 0 | 0 | 0 | 0 |  |  |
| No trespassing / beware of dogs | N | 18.6% | 0 | 2 | 0 | 0 | 0 | 0 | 0 | 1.50 | (N) |
| Security warning | N | 7.6% | 1 | 0 | 0 | 0 | 0 | 0 | 0 |  |  |
| **Social Environment** |  |  |  |  |  |  |  |  |  |  |  |
| Children present in the street | P | 5.9% | 1 | 0 | 0 | 0 | 0 | 0 | 0 | 3.00 | (P) |
| Children engaged in active behavior | P | 5.9% | 1 | 0 | 0 | 0 | 0 | 0 | 0 |  |  |
| Teenagers/adults present in the street | P | 85.6% | 0 | 0 | 0 | 0 | 0 | 6 | 0 |  |  |
| Teenagers/adults engaged in active behavior | P | 81.4% | 0 | 0 | 0 | 0 | 0 | 6 | 0 |  |  |
| Older adults (>65 years) present in the street | P | 17.8% | 0 | 2 | 0 | 0 | 0 | 0 | 0 |  |  |
| Older adults (>65 years) engaged in active behavior | P | 16.1% | 0 | 2 | 0 | 0 | 0 | 0 | 0 |  |  |
| Stray dogs or animals (not squirrels or rabbits) | N | 0.8% | 1 | 0 | 0 | 0 | 0 | 0 | 0 | 1.00 | (N) |

Abbreviations: TRAIN, Travel Related Activity in Neighborhoods.

a. Observed features were arranged into two classes: positive (P) and negative (N). This classification was based on the expected direction of the relationship between each feature and physical activity behavior, as evidenced by findings in available literature.

b. C.I. scores were calculated separately for each class (positive vs. negative) in each domain. To calculate a C.I. score, we first dichotomized the observed categories for selected features, such that features with multiple categories were collapsed into “any observation” versus “no observation. Thereafter, we calculated the percentage of all the audited segments in the entire study area with “any observation” of these selected features. We then converted the calculated percentage to seven descriptive statements that ranged from “hardly ever” to “always”. For example, a feature that was observed in 0-10.9 percent of the audited segments was labeled "hardly ever," and so on. The seven descriptive statements were thereafter assigned scores, ranging from 1.00 for "hardly ever" (0%-10.9%) to 7.00 for "always" (91%-100%). We then calculated the average score for that class and maintained the min=1.00, max=7.00 average score (i.e., the C.I. score). Where positive relationship with physical activity is expected, a high C.I. score is indicative of prevalence of physical activity friendly features, whereas, for negative relationships, a high score indicates prevalence non-physical activity friendly features.

c. Data shown (percentages) are based on a subset of the audited 590 segments that are applicable to these particular audit questions. For example. Sidewalk continuity only applies to segments with sidewalks.

**Table B6.** Calculating the Composite Index (C.I.) scores for the **Second Ward** super neighborhood (78 segments). Houston TRAIN Study, 2014.

| **Descriptive statements** | | | Hardly ever | Rarely | Occasionally | Half the time | Frequently | Usually | Always | Composite Index^b^ | |
| --- | --- | --- | --- | --- | --- | --- | --- | --- | --- | --- | --- |
| **Percent ranges** | | | 0-10.9 | 11-30.9 | 31-45.9 | 46-55.9 | 56-70.9 | 71-90.9 | 91-100 |  |  |
| **Scores** | | | 1.00 | 2.00 | 3.00 | 4.00 | 5.00 | 6.00 | 7.00 |  |  |
|  | P/N^a^ | (%) |  |  |  |  |  |  |  |  | |
| **Land Use** |  |  |  |  |  |  |  |  |  |  |  |
| Land use integration | P | 35.9% | 0 | 0 | 3 | 0 | 0 | 0 | 0 | 1.25 | (P) |
| Office building | P | 5.1% | 1 | 0 | 0 | 0 | 0 | 0 | 0 |  |  |
| Other services – e.g. beautician and lawyer | P | 10.3% | 1 | 0 | 0 | 0 | 0 | 0 | 0 |  |  |
| Fast food restaurants | P | 5.1% | 1 | 0 | 0 | 0 | 0 | 0 | 0 |  |  |
| Strip mall | P | 0.0% | 1 | 0 | 0 | 0 | 0 | 0 | 0 |  |  |
| Transportation facility | P | 5.1% | 1 | 0 | 0 | 0 | 0 | 0 | 0 |  |  |
| Place of worship | P | 3.8% | 1 | 0 | 0 | 0 | 0 | 0 | 0 |  |  |
| Park | P | 0.0% | 1 | 0 | 0 | 0 | 0 | 0 | 0 |  |  |
| Warehouses, factories, or industrial buildings | N | 24.4% | 0 | 2 | 0 | 0 | 0 | 0 | 0 | 3.50 | (N) |
| Auto shop | N | 19.2% | 0 | 2 | 0 | 0 | 0 | 0 | 0 |  |  |
| Parking lot or parking garage | N | 44.9% | 0 | 0 | 3 | 0 | 0 | 0 | 0 |  |  |
| Driveway (either residential or non-residential) | N | 93.6% | 0 | 0 | 0 | 0 | 0 | 0 | 7 |  |  |
| Abandoned building or vacant lot | N | 56.4% | 0 | 0 | 0 | 0 | 5 | 0 | 0 |  |  |
| Major transportation development (e.g. bridge, tunnel) | N | 15.4% | 0 | 2 | 0 | 0 | 0 | 0 | 0 |  |  |
| **Transportation** |  |  |  |  |  |  |  |  |  |  |  |
| Any alternative transportation modes | P | 89.7% | 0 | 0 | 0 | 0 | 0 | 6 | 0 | 3.83 | (P) |
| Any sidewalk facility | P | 92.3% | 0 | 0 | 0 | 0 | 0 | 0 | 7 |  |  |
| Sidewalk continuity; at least 1 side of street (both ends)^c^ | P | 58.3% | 0 | 0 | 0 | 0 | 5 | 0 | 0 |  |  |
| Curvilinear curbs; at least 1 side of street (both ends)**^c^** | P | 40.3% | 0 | 0 | 3 | 0 | 0 | 0 | 0 |  |  |
| Sidewalk coverage on left side of segment (>75%)^c^ | P | 63.9% | 0 | 0 | 0 | 0 | 5 | 0 | 0 |  |  |
| Sidewalk coverage on right side of segment (>75%)^c^ | P | 68.1% | 0 | 0 | 0 | 0 | 5 | 0 | 0 |  |  |
| Bike lane or marked shoulder (for bikes) | P | 2.6% | 1 | 0 | 0 | 0 | 0 | 0 | 0 |  |  |
| Bike racks present | P | 0.0% | 1 | 0 | 0 | 0 | 0 | 0 | 0 |  |  |
| Bus/transit stop present | P | 16.7% | 0 | 2 | 0 | 0 | 0 | 0 | 0 |  |  |
| Bus stop; covered shelter with/out bench^c^ | P | 23.1% | 0 | 2 | 0 | 0 | 0 | 0 | 0 |  |  |
| Non-concrete multi-use trails or paths | P | 11.5% | 0 | 2 | 0 | 0 | 0 | 0 | 0 |  |  |
| Posted speed limit | P | 10.3% | 1 | 0 | 0 | 0 | 0 | 0 | 0 |  |  |
| On-street parking | P | 34.6% | 0 | 0 | 3 | 0 | 0 | 0 | 0 |  |  |
| >3 directions at intersections | P | 84.6% | 0 | 0 | 0 | 0 | 0 | 6 | 0 |  |  |
| Road design to reduce car volume/speed | P | 20.5% | 0 | 2 | 0 | 0 | 0 | 0 | 0 |  |  |
| Traffic calming devices to reduce car volume/speed | P | 80.8% | 0 | 0 | 0 | 0 | 0 | 6 | 0 |  |  |
| Crossing aids for pedestrian / bicyclist | P | 57.7% | 0 | 0 | 0 | 0 | 5 | 0 | 0 |  |  |
| Street lighting for sidewalk, street shoulders, etc. | P | 93.6% | 0 | 0 | 0 | 0 | 0 | 0 | 7 |  |  |
| Sidewalk has heaves, cracks, broken sections, etc.^c^ | N | 44.4% | 0 | 0 | 3 | 0 | 0 | 0 | 0 | 3.50 | (N) |
| Sidewalk blocked by obstacles^c^ | N | 51.4% | 0 | 0 | 0 | 4 | 0 | 0 | 0 |  |  |
| **Facilities** |  |  |  |  |  |  |  |  |  |  |  |
| Public / recreational facilities | P | 1.3% | 1 | 0 | 0 | 0 | 0 | 0 | 0 | 1.00 | (P) |
| Public / recreational equipment | P | 1.3% | 1 | 0 | 0 | 0 | 0 | 0 | 0 |  |  |
| Playground equipment | P | 0.0% | 1 | 0 | 0 | 0 | 0 | 0 | 0 |  |  |
| **Aesthetics** |  |  |  |  |  |  |  |  |  |  |  |
| Attractive features (e.g., architecture, vegetation) | P | 52.6% | 0 | 0 | 0 | 4 | 0 | 0 | 0 | 5.00 | (P) |
| Comfort features (shade, trees, benches, etc.) | P | 80.8% | 0 | 0 | 0 | 0 | 0 | 6 | 0 |  |  |
| Air pollution | N | 17.9% | 0 | 2 | 0 | 0 | 0 | 0 | 0 | 4.17 | (N) |
| Noise pollution | N | 26.9% | 0 | 2 | 0 | 0 | 0 | 0 | 0 |  |  |
| Physical disorder (general) | N | 85.9% | 0 | 0 | 0 | 0 | 0 | 6 | 0 |  |  |
| Whole or broken beer or liquor bottles or can | N | 47.4% | 0 | 0 | 0 | 4 | 0 | 0 | 0 |  |  |
| Cigarette, cigar butts or discarded cigarette packages | N | 62.8% | 0 | 0 | 0 | 0 | 5 | 0 | 0 |  |  |
| Garbage, litter, or broken glass | N | 80.8% | 0 | 0 | 0 | 0 | 0 | 6 | 0 |  |  |
| **Signage** |  |  |  |  |  |  |  |  |  |  |  |
| Pedestrian or bicyclist friendly traffic signs | P | 48.7% | 0 | 0 | 0 | 4 | 0 | 0 | 0 | 1.60 | (P) |
| Share the road sign | P | 5.1% | 1 | 0 | 0 | 0 | 0 | 0 | 0 |  |  |
| Religious message | P | 3.8% | 1 | 0 | 0 | 0 | 0 | 0 | 0 |  |  |
| Political message | P | 7.7% | 1 | 0 | 0 | 0 | 0 | 0 | 0 |  |  |
| Fast food billboard | P | 5.1% | 1 | 0 | 0 | 0 | 0 | 0 | 0 |  |  |
| No trespassing / beware of dogs | N | 32.1% | 0 | 0 | 3 | 0 | 0 | 0 | 0 | 2.50 | (N) |
| Security warning | N | 16.7% | 0 | 2 | 0 | 0 | 0 | 0 | 0 |  |  |
| **Social Environment** |  |  |  |  |  |  |  |  |  |  |  |
| Children present in the street | P | 9.0% | 1 | 0 | 0 | 0 | 0 | 0 | 0 | 2.17 | (P) |
| Children engaged in active behavior | P | 6.4% | 1 | 0 | 0 | 0 | 0 | 0 | 0 |  |  |
| Teenagers/adults present in the street | P | 61.5% | 0 | 0 | 0 | 0 | 5 | 0 | 0 |  |  |
| Teenagers/adults engaged in active behavior | P | 46.2% | 0 | 0 | 0 | 4 | 0 | 0 | 0 |  |  |
| Older adults (>65 years) present in the street | P | 10.3% | 1 | 0 | 0 | 0 | 0 | 0 | 0 |  |  |
| Older adults (>65 years) engaged in active behavior | P | 9.0% | 1 | 0 | 0 | 0 | 0 | 0 | 0 |  |  |
| Stray dogs or animals (not squirrels or rabbits) | N | 20.5% | 0 | 2 | 0 | 0 | 0 | 0 | 0 | 2.00 | (N) |

Abbreviations: TRAIN, Travel Related Activity in Neighborhoods.

a. Observed features were arranged into two classes: positive (P) and negative (N). This classification was based on the expected direction of the relationship between each feature and physical activity behavior, as evidenced by findings in available literature.

b. C.I. scores were calculated separately for each class (positive vs. negative) in each domain. To calculate a C.I. score, we first dichotomized the observed categories for selected features, such that features with multiple categories were collapsed into “any observation” versus “no observation. Thereafter, we calculated the percentage of all the audited segments in the entire study area with “any observation” of these selected features. We then converted the calculated percentage to seven descriptive statements that ranged from “hardly ever” to “always”. For example, a feature that was observed in 0-10.9 percent of the audited segments was labeled "hardly ever," and so on. The seven descriptive statements were thereafter assigned scores, ranging from 1.00 for "hardly ever" (0%-10.9%) to 7.00 for "always" (91%-100%). We then calculated the average score for that class and maintained the min=1.00, max=7.00 average score (i.e., the C.I. score). Where positive relationship with physical activity is expected, a high C.I. score is indicative of prevalence of physical activity friendly features, whereas, for negative relationships, a high score indicates prevalence non-physical activity friendly features.

c. Data shown (percentages) are based on a subset of the audited 590 segments that are applicable to these particular audit questions. For example. Sidewalk continuity only applies to segments with sidewalks.

**Table B7.** Calculating the Composite Index (C.I.) scores for the **Magnolia** super neighborhood (36 segments). Houston TRAIN Study, 2014.

| **Descriptive statements** | | | Hardly ever | Rarely | Occasionally | Half the time | Frequently | Usually | Always | Composite Index^b^ | | |
| --- | --- | --- | --- | --- | --- | --- | --- | --- | --- | --- | --- | --- |
| **Percent ranges** | | | 0-10.9 | 11-30.9 | 31-45.9 | 46-55.9 | 56-70.9 | 71-90.9 | 91-100 |  |  |  |
| **Scores** | | | 1.00 | 2.00 | 3.00 | 4.00 | 5.00 | 6.00 | 7.00 |  |  |  |
|  | P/N^a^ | (%) |  |  |  |  |  |  |  |  | | |
| **Land Use** |  |  |  |  |  |  |  |  |  |  |  |  |
| Land use integration | P | 33.3% | 0 | 0 | 3 | 0 | 0 | 0 | 0 | 1.63 | (P) |  |
| Office building | P | 8.3% | 1 | 0 | 0 | 0 | 0 | 0 | 0 |  |  |  |
| Other services – e.g. beautician and lawyer | P | 30.6% | 0 | 2 | 0 | 0 | 0 | 0 | 0 |  |  |  |
| Fast food restaurants | P | 2.8% | 1 | 0 | 0 | 0 | 0 | 0 | 0 |  |  |  |
| Strip mall | P | 16.7% | 0 | 2 | 0 | 0 | 0 | 0 | 0 |  |  |  |
| Transportation facility | P | 13.9% | 0 | 2 | 0 | 0 | 0 | 0 | 0 |  |  |  |
| Place of worship | P | 2.8% | 1 | 0 | 0 | 0 | 0 | 0 | 0 |  |  |  |
| Park | P | 0.0% | 1 | 0 | 0 | 0 | 0 | 0 | 0 |  |  |  |
| Warehouses, factories, or industrial buildings | N | 16.7% | 0 | 2 | 0 | 0 | 0 | 0 | 0 | 3.67 | (N) |  |
| Auto shop | N | 19.4% | 0 | 2 | 0 | 0 | 0 | 0 | 0 |  |  |  |
| Parking lot or parking garage | N | 72.2% | 0 | 0 | 0 | 0 | 0 | 6 | 0 |  |  |  |
| Driveway (either residential or non-residential) | N | 94.4% | 0 | 0 | 0 | 0 | 0 | 0 | 7 |  |  |  |
| Abandoned building or vacant lot | N | 36.1% | 0 | 0 | 3 | 0 | 0 | 0 | 0 |  |  |  |
| Major transportation development (e.g. bridge, tunnel) | N | 20.7% | 0 | 2 | 0 | 0 | 0 | 0 | 0 |  |  |  |
| **Transportation** |  |  |  |  |  |  |  |  |  |  |  |  |
| Any alternative transportation modes | P | 86.1% | 0 | 0 | 0 | 0 | 0 | 6 | 0 | 4.06 | (P) |  |
| Any sidewalk facility | P | 86.1% | 0 | 0 | 0 | 0 | 0 | 6 | 0 |  |  |  |
| Sidewalk continuity; at least 1 side of street (both ends)^c^ | P | 67.7% | 0 | 0 | 0 | 0 | 5 | 0 | 0 |  |  |  |
| Curvilinear curbs; at least 1 side of street (both ends)**^c^** | P | 58.1% | 0 | 0 | 0 | 0 | 5 | 0 | 0 |  |  |  |
| Sidewalk coverage on left side of segment (>75%)^c^ | P | 80.6% | 0 | 0 | 0 | 0 | 0 | 6 | 0 |  |  |  |
| Sidewalk coverage on right side of segment (>75%)^c^ | P | 87.1% | 0 | 0 | 0 | 0 | 0 | 6 | 0 |  |  |  |
| Bike lane or marked shoulder (for bikes) | P | 8.3% | 1 | 0 | 0 | 0 | 0 | 0 | 0 |  |  |  |
| Bike racks present | P | 2.8% | 1 | 0 | 0 | 0 | 0 | 0 | 0 |  |  |  |
| Bus/transit stop present | P | 27.8% | 0 | 2 | 0 | 0 | 0 | 0 | 0 |  |  |  |
| Bus stop; covered shelter with/out bench^c^ | P | 70.0% | 0 | 0 | 0 | 0 | 5 | 0 | 0 |  |  |  |
| Non-concrete multi-use trails or paths | P | 0.0% | 1 | 0 | 0 | 0 | 0 | 0 | 0 |  |  |  |
| Posted speed limit | P | 2.8% | 1 | 0 | 0 | 0 | 0 | 0 | 0 |  |  |  |
| On-street parking | P | 22.2% | 0 | 2 | 0 | 0 | 0 | 0 | 0 |  |  |  |
| >3 directions at intersections | P | 88.9% | 0 | 0 | 0 | 0 | 0 | 6 | 0 |  |  |  |
| Road design to reduce car volume/speed | P | 25.0% | 0 | 2 | 0 | 0 | 0 | 0 | 0 |  |  |  |
| Traffic calming devices to reduce car volume/speed | P | 80.6% | 0 | 0 | 0 | 0 | 0 | 6 | 0 |  |  |  |
| Crossing aids for pedestrian / bicyclist | P | 61.1% | 0 | 0 | 0 | 0 | 5 | 0 | 0 |  |  |  |
| Street lighting for sidewalk, street shoulders, etc. | P | 100.0% | 0 | 0 | 0 | 0 | 0 | 0 | 7 |  |  |  |
| Sidewalk has heaves, cracks, broken sections, etc.^c^ | N | 61.3% | 0 | 0 | 0 | 0 | 5 | 0 | 0 | 4.00 | (N) |  |
| Sidewalk blocked by obstacles^c^ | N | 35.5% | 0 | 0 | 3 | 0 | 0 | 0 | 0 |  |  |  |
| **Facilities** |  |  |  |  |  |  |  |  |  |  |  |  |
| Public / recreational facilities | P | 0.0% | 1 | 0 | 0 | 0 | 0 | 0 | 0 | 1.00 | (P) |  |
| Public / recreational equipment | P | 2.8% | 1 | 0 | 0 | 0 | 0 | 0 | 0 |  |  |  |
| Playground equipment | P | 0.0% | 1 | 0 | 0 | 0 | 0 | 0 | 0 |  |  |  |
| **Aesthetics** |  |  |  |  |  |  |  |  |  |  |  |  |
| Attractive features (e.g., architecture, vegetation) | P | 41.7% | 0 | 0 | 3 | 0 | 0 | 0 | 0 | 4.00 | (P) |  |
| Comfort features (shade, trees, benches, etc.) | P | 69.4% | 0 | 0 | 0 | 0 | 5 | 0 | 0 |  |  |  |
| Air pollution | N | 2.8% | 1 | 0 | 0 | 0 | 0 | 0 | 0 | 4.83 | (N) |  |
| Noise pollution | N | 44.4% | 0 | 0 | 3 | 0 | 0 | 0 | 0 |  |  |  |
| Physical disorder (general) | N | 97.2% | 0 | 0 | 0 | 0 | 0 | 0 | 7 |  |  |  |
| Whole or broken beer or liquor bottles or can | N | 55.6% | 0 | 0 | 0 | 4 | 0 | 0 | 0 |  |  |  |
| Cigarette, cigar butts or discarded cigarette packages | N | 94.4% | 0 | 0 | 0 | 0 | 0 | 0 | 7 |  |  |  |
| Garbage, litter, or broken glass | N | 91.7% | 0 | 0 | 0 | 0 | 0 | 0 | 7 |  |  |  |
| **Signage** |  |  |  |  |  |  |  |  |  |  |  |  |
| Pedestrian or bicyclist friendly traffic signs | P | 52.8% | 0 | 0 | 0 | 4 | 0 | 0 | 0 | 1.80 | (P) |  |
| Share the road sign | P | 2.8% | 1 | 0 | 0 | 0 | 0 | 0 | 0 |  |  |  |
| Religious message | P | 2.8% | 1 | 0 | 0 | 0 | 0 | 0 | 0 |  |  |  |
| Political message | P | 8.3% | 1 | 0 | 0 | 0 | 0 | 0 | 0 |  |  |  |
| Fast food billboard | P | 19.4% | 0 | 2 | 0 | 0 | 0 | 0 | 0 |  |  |  |
| No trespassing / beware of dogs | N | 38.9% | 0 | 0 | 3 | 0 | 0 | 0 | 0 | 2.50 | (N) |  |
| Security warning | N | 22.2% | 0 | 2 | 0 | 0 | 0 | 0 | 0 |  |  |  |
| **Social Environment** |  |  |  |  |  |  |  |  |  |  |  |  |
| Children present in the street | P | 2.8% | 1 | 0 | 0 | 0 | 0 | 0 | 0 | 2.50 | (P) |  |
| Children engaged in active behavior | P | 2.8% | 1 | 0 | 0 | 0 | 0 | 0 | 0 |  |  |  |
| Teenagers/adults present in the street | P | 83.3% | 0 | 0 | 0 | 0 | 0 | 6 | 0 |  |  |  |
| Teenagers/adults engaged in active behavior | P | 66.7% | 0 | 0 | 0 | 0 | 5 | 0 | 0 |  |  |  |
| Older adults (>65 years) present in the street | P | 8.3% | 1 | 0 | 0 | 0 | 0 | 0 | 0 |  |  |  |
| Older adults (>65 years) engaged in active behavior | P | 8.3% | 1 | 0 | 0 | 0 | 0 | 0 | 0 |  |  |  |
| Stray dogs or animals (not squirrels or rabbits) | N | 0.0% | 1 | 0 | 0 | 0 | 0 | 0 | 0 | 1.00 | (N) |  |

Abbreviations: TRAIN, Travel Related Activity in Neighborhoods.

a. Observed features were arranged into two classes: positive (P) and negative (N). This classification was based on the expected direction of the relationship between each feature and physical activity behavior, as evidenced by findings in available literature.

b. C.I. scores were calculated separately for each class (positive vs. negative) in each domain. To calculate a C.I. score, we first dichotomized the observed categories for selected features, such that features with multiple categories were collapsed into “any observation” versus “no observation. Thereafter, we calculated the percentage of all the audited segments in the entire study area with “any observation” of these selected features. We then converted the calculated percentage to seven descriptive statements that ranged from “hardly ever” to “always”. For example, a feature that was observed in 0-10.9 percent of the audited segments was labeled "hardly ever," and so on. The seven descriptive statements were thereafter assigned scores, ranging from 1.00 for "hardly ever" (0%-10.9%) to 7.00 for "always" (91%-100%). We then calculated the average score for that class and maintained the min=1.00, max=7.00 average score (i.e., the C.I. score). Where positive relationship with physical activity is expected, a high C.I. score is indicative of prevalence of physical activity friendly features, whereas, for negative relationships, a high score indicates prevalence non-physical activity friendly features.

c. Data shown (percentages) are based on a subset of the audited 590 segments that are applicable to these particular audit questions. For example. Sidewalk continuity only applies to segments with sidewalks.

**Table B8.** Calculating the Composite Index (C.I.) scores for the **Greater Eastwood** super neighborhood (14 segments). Houston TRAIN Study, 2014.

| **Descriptive statements** | | | Hardly ever | Rarely | Occasionally | Half the time | Frequently | Usually | Always | Composite Index^b^ | |
| --- | --- | --- | --- | --- | --- | --- | --- | --- | --- | --- | --- |
| **Percent ranges** | | | 0-10.9 | 11-30.9 | 31-45.9 | 46-55.9 | 56-70.9 | 71-90.9 | 91-100 |  |  |
| **Scores** | | | 1.00 | 2.00 | 3.00 | 4.00 | 5.00 | 6.00 | 7.00 |  |  |
|  | P/N^a^ | (%) |  |  |  |  |  |  |  |  | |
| **Land Use** |  |  |  |  |  |  |  |  |  |  |  |
| Land use integration | P | 21.4% | 0 | 2 | 0 | 0 | 0 | 0 | 0 | 1.50 | (P) |
| Office building | P | 14.3% | 0 | 2 | 0 | 0 | 0 | 0 | 0 |  |  |
| Other services – e.g. beautician and lawyer | P | 21.4% | 0 | 2 | 0 | 0 | 0 | 0 | 0 |  |  |
| Fast food restaurants | P | 0.0% | 1 | 0 | 0 | 0 | 0 | 0 | 0 |  |  |
| Strip mall | P | 0.0% | 1 | 0 | 0 | 0 | 0 | 0 | 0 |  |  |
| Transportation facility | P | 21.4% | 0 | 2 | 0 | 0 | 0 | 0 | 0 |  |  |
| Place of worship | P | 0.0% | 1 | 0 | 0 | 0 | 0 | 0 | 0 |  |  |
| Park | P | 0.0% | 1 | 0 | 0 | 0 | 0 | 0 | 0 |  |  |
| Warehouses, factories, or industrial buildings | N | 21.4% | 0 | 2 | 0 | 0 | 0 | 0 | 0 | 3.33 | (N) |
| Auto shop | N | 21.4% | 0 | 2 | 0 | 0 | 0 | 0 | 0 |  |  |
| Parking lot or parking garage | N | 28.6% | 0 | 2 | 0 | 0 | 0 | 0 | 0 |  |  |
| Driveway (either residential or non-residential) | N | 92.9% | 0 | 0 | 0 | 0 | 0 | 0 | 7 |  |  |
| Abandoned building or vacant lot | N | 64.3% | 0 | 0 | 0 | 0 | 5 | 0 | 0 |  |  |
| Major transportation development (e.g. bridge, tunnel) | N | 21.4% | 0 | 2 | 0 | 0 | 0 | 0 | 0 |  |  |
| **Transportation** |  |  |  |  |  |  |  |  |  |  |  |
| Any alternative transportation modes | P | 92.9% | 0 | 0 | 0 | 0 | 0 | 0 | 7 | 4.17 | (P) |
| Any sidewalk facility | P | 92.9% | 0 | 0 | 0 | 0 | 0 | 0 | 7 |  |  |
| Sidewalk continuity; at least 1 side of street (both ends)^c^ | P | 53.8% | 0 | 0 | 0 | 4 | 0 | 0 | 0 |  |  |
| Curvilinear curbs; at least 1 side of street (both ends)**^c^** | P | 61.5% | 0 | 0 | 0 | 0 | 5 | 0 | 0 |  |  |
| Sidewalk coverage on left side of segment (>75%)^c^ | P | 84.6% | 0 | 0 | 0 | 0 | 0 | 6 | 0 |  |  |
| Sidewalk coverage on right side of segment (>75%)^c^ | P | 61.5% | 0 | 0 | 0 | 0 | 5 | 0 | 0 |  |  |
| Bike lane or marked shoulder (for bikes) | P | 7.1% | 1 | 0 | 0 | 0 | 0 | 0 | 0 |  |  |
| Bike racks present | P | 0.0% | 1 | 0 | 0 | 0 | 0 | 0 | 0 |  |  |
| Bus/transit stop present | P | 28.6% | 0 | 2 | 0 | 0 | 0 | 0 | 0 |  |  |
| Bus stop; covered shelter with/out bench^c^ | P | 75.0% | 0 | 0 | 0 | 0 | 0 | 6 | 0 |  |  |
| Non-concrete multi-use trails or paths | P | 0.0% | 1 | 0 | 0 | 0 | 0 | 0 | 0 |  |  |
| Posted speed limit | P | 7.1% | 1 | 0 | 0 | 0 | 0 | 0 | 0 |  |  |
| On-street parking | P | 21.4% | 0 | 2 | 0 | 0 | 0 | 0 | 0 |  |  |
| >3 directions at intersections | P | 57.1% | 0 | 0 | 0 | 0 | 5 | 0 | 0 |  |  |
| Road design to reduce car volume/speed | P | 57.1% | 0 | 0 | 0 | 0 | 5 | 0 | 0 |  |  |
| Traffic calming devices to reduce car volume/speed | P | 78.6% | 0 | 0 | 0 | 0 | 0 | 6 | 0 |  |  |
| Crossing aids for pedestrian / bicyclist | P | 50.0% | 0 | 0 | 0 | 4 | 0 | 0 | 0 |  |  |
| Street lighting for sidewalk, street shoulders, etc. | P | 100.0% | 0 | 0 | 0 | 0 | 0 | 0 | 7 |  |  |
| Sidewalk has heaves, cracks, broken sections, etc.^c^ | N | 38.5% | 0 | 0 | 3 | 0 | 0 | 0 | 0 | 3.50 | (N) |
| Sidewalk blocked by obstacles^c^ | N | 46.2% | 0 | 0 | 0 | 4 | 0 | 0 | 0 |  |  |
| **Facilities** |  |  |  |  |  |  |  |  |  |  |  |
| Public / recreational facilities | P | 0.0% | 1 | 0 | 0 | 0 | 0 | 0 | 0 | 1.00 | (P) |
| Public / recreational equipment | P | 0.0% | 1 | 0 | 0 | 0 | 0 | 0 | 0 |  |  |
| Playground equipment | P | 0.0% | 1 | 0 | 0 | 0 | 0 | 0 | 0 |  |  |
| **Aesthetics** |  |  |  |  |  |  |  |  |  |  |  |
| Attractive features (e.g., architecture, vegetation) | P | 50.0% | 0 | 0 | 0 | 4 | 0 | 0 | 0 | 5.00 | (P) |
| Comfort features (shade, trees, benches, etc.) | P | 78.6% | 0 | 0 | 0 | 0 | 0 | 6 | 0 |  |  |
| Air pollution | N | 7.1% | 1 | 0 | 0 | 0 | 0 | 0 | 0 | 4.50 | (N) |
| Noise pollution | N | 35.7% | 0 | 0 | 3 | 0 | 0 | 0 | 0 |  |  |
| Physical disorder (general) | N | 92.9% | 0 | 0 | 0 | 0 | 0 | 0 | 7 |  |  |
| Whole or broken beer or liquor bottles or can | N | 71.4% | 0 | 0 | 0 | 0 | 0 | 6 | 0 |  |  |
| Cigarette, cigar butts or discarded cigarette packages | N | 85.7% | 0 | 0 | 0 | 0 | 0 | 6 | 0 |  |  |
| Garbage, litter, or broken glass | N | 50.0% | 0 | 0 | 0 | 4 | 0 | 0 | 0 |  |  |
| **Signage** |  |  |  |  |  |  |  |  |  |  |  |
| Pedestrian or bicyclist friendly traffic signs | P | 42.9% | 0 | 0 | 3 | 0 | 0 | 0 | 0 | 1.40 | (P) |
| Share the road sign | P | 0.0% | 1 | 0 | 0 | 0 | 0 | 0 | 0 |  |  |
| Religious message | P | 0.0% | 1 | 0 | 0 | 0 | 0 | 0 | 0 |  |  |
| Political message | P | 0.0% | 1 | 0 | 0 | 0 | 0 | 0 | 0 |  |  |
| Fast food billboard | P | 0.0% | 1 | 0 | 0 | 0 | 0 | 0 | 0 |  |  |
| No trespassing / beware of dogs | N | 35.7% | 0 | 0 | 3 | 0 | 0 | 0 | 0 | 2.00 | (N) |
| Security warning | N | 7.1% | 1 | 0 | 0 | 0 | 0 | 0 | 0 |  |  |
| **Social Environment** |  |  |  |  |  |  |  |  |  |  |  |
| Children present in the street | P | 0.0% | 1 | 0 | 0 | 0 | 0 | 0 | 0 | 2.17 | (P) |
| Children engaged in active behavior | P | 0.0% | 1 | 0 | 0 | 0 | 0 | 0 | 0 |  |  |
| Teenagers/adults present in the street | P | 71.4% | 0 | 0 | 0 | 0 | 0 | 6 | 0 |  |  |
| Teenagers/adults engaged in active behavior | P | 35.7% | 0 | 0 | 3 | 0 | 0 | 0 | 0 |  |  |
| Older adults (>65 years) present in the street | P | 7.1% | 1 | 0 | 0 | 0 | 0 | 0 | 0 |  |  |
| Older adults (>65 years) engaged in active behavior | P | 0.0% | 1 | 0 | 0 | 0 | 0 | 0 | 0 |  |  |
| Stray dogs or animals (not squirrels or rabbits) | N | 35.7% | 0 | 0 | 3 | 0 | 0 | 0 | 0 | 3.00 | (N) |

Abbreviations: TRAIN, Travel Related Activity in Neighborhoods.

a. Observed features were arranged into two classes: positive (P) and negative (N). This classification was based on the expected direction of the relationship between each feature and physical activity behavior, as evidenced by findings in available literature.

b. C.I. scores were calculated separately for each class (positive vs. negative) in each domain. To calculate a C.I. score, we first dichotomized the observed categories for selected features, such that features with multiple categories were collapsed into “any observation” versus “no observation. Thereafter, we calculated the percentage of all the audited segments in the entire study area with “any observation” of these selected features. We then converted the calculated percentage to seven descriptive statements that ranged from “hardly ever” to “always”. For example, a feature that was observed in 0-10.9 percent of the audited segments was labeled "hardly ever," and so on. The seven descriptive statements were thereafter assigned scores, ranging from 1.00 for "hardly ever" (0%-10.9%) to 7.00 for "always" (91%-100%). We then calculated the average score for that class and maintained the min=1.00, max=7.00 average score (i.e., the C.I. score). Where positive relationship with physical activity is expected, a high C.I. score is indicative of prevalence of physical activity friendly features, whereas, for negative relationships, a high score indicates prevalence non-physical activity friendly features.

c. Data shown (percentages) are based on a subset of the audited 590 segments that are applicable to these particular audit questions. For example. Sidewalk continuity only applies to segments with sidewalks.

**Table B9.** Calculating the Composite Index (C.I.) scores for the **Greater Third Ward** super neighborhood (42 segments). Houston TRAIN Study, 2014.

| **Descriptive statements** | | | Hardly ever | Rarely | Occasionally | Half the time | Frequently | Usually | Always | Composite Index^b^ | |
| --- | --- | --- | --- | --- | --- | --- | --- | --- | --- | --- | --- |
| **Percent ranges** | | | 0-10.9 | 11-30.9 | 31-45.9 | 46-55.9 | 56-70.9 | 71-90.9 | 91-100 |  |  |
| **Scores** | | | 1.00 | 2.00 | 3.00 | 4.00 | 5.00 | 6.00 | 7.00 |  |  |
|  | P/N^a^ | (%) |  |  |  |  |  |  |  |  | |
| **Land Use** |  |  |  |  |  |  |  |  |  |  |  |
| Land use integration | P | 14.3% | 0 | 2 | 0 | 0 | 0 | 0 | 0 | 1.25 | (P) |
| Office building | P | 0.0% | 1 | 0 | 0 | 0 | 0 | 0 | 0 |  |  |
| Other services – e.g. beautician and lawyer | P | 2.4% | 1 | 0 | 0 | 0 | 0 | 0 | 0 |  |  |
| Fast food restaurants | P | 14.3% | 0 | 2 | 0 | 0 | 0 | 0 | 0 |  |  |
| Strip mall | P | 4.8% | 1 | 0 | 0 | 0 | 0 | 0 | 0 |  |  |
| Transportation facility | P | 0.0% | 1 | 0 | 0 | 0 | 0 | 0 | 0 |  |  |
| Place of worship | P | 9.5% | 1 | 0 | 0 | 0 | 0 | 0 | 0 |  |  |
| Park | P | 2.4% | 1 | 0 | 0 | 0 | 0 | 0 | 0 |  |  |
| Warehouses, factories, or industrial buildings | N | 4.8% | 1 | 0 | 0 | 0 | 0 | 0 | 0 | 2.67 | (N) |
| Auto shop | N | 4.8% | 1 | 0 | 0 | 0 | 0 | 0 | 0 |  |  |
| Parking lot or parking garage | N | 40.5% | 0 | 0 | 3 | 0 | 0 | 0 | 0 |  |  |
| Driveway (either residential or non-residential) | N | 92.9% | 0 | 0 | 0 | 0 | 0 | 0 | 7 |  |  |
| Abandoned building or vacant lot | N | 45.2% | 0 | 0 | 3 | 0 | 0 | 0 | 0 |  |  |
| Major transportation development (e.g. bridge, tunnel) | N | 7.1% | 1 | 0 | 0 | 0 | 0 | 0 | 0 |  |  |
| **Transportation** |  |  |  |  |  |  |  |  |  |  |  |
| Any alternative transportation modes | P | 57.1% | 0 | 0 | 0 | 0 | 5 | 0 | 0 | 4.06 | (P) |
| Any sidewalk facility | P | 57.1% | 0 | 0 | 0 | 0 | 5 | 0 | 0 |  |  |
| Sidewalk continuity; at least 1 side of street (both ends)^c^ | P | 45.8% | 0 | 0 | 3 | 0 | 0 | 0 | 0 |  |  |
| Curvilinear curbs; at least 1 side of street (both ends)**^c^** | P | 79.2% | 0 | 0 | 0 | 0 | 0 | 6 | 0 |  |  |
| Sidewalk coverage on left side of segment (>75%)^c^ | P | 75.0% | 0 | 0 | 0 | 0 | 0 | 6 | 0 |  |  |
| Sidewalk coverage on right side of segment (>75%)^c^ | P | 66.7% | 0 | 0 | 0 | 0 | 5 | 0 | 0 |  |  |
| Bike lane or marked shoulder (for bikes) | P | 0.0% | 1 | 0 | 0 | 0 | 0 | 0 | 0 |  |  |
| Bike racks present | P | 2.4% | 1 | 0 | 0 | 0 | 0 | 0 | 0 |  |  |
| Bus/transit stop present | P | 21.4% | 0 | 2 | 0 | 0 | 0 | 0 | 0 |  |  |
| Bus stop; covered shelter with/out bench^c^ | P | 77.8% | 0 | 0 | 0 | 0 | 0 | 6 | 0 |  |  |
| Non-concrete multi-use trails or paths | P | 2.4% | 1 | 0 | 0 | 0 | 0 | 0 | 0 |  |  |
| Posted speed limit | P | 11.9% | 0 | 2 | 0 | 0 | 0 | 0 | 0 |  |  |
| On-street parking | P | 31.0% | 0 | 0 | 3 | 0 | 0 | 0 | 0 |  |  |
| >3 directions at intersections | P | 90.5% | 0 | 0 | 0 | 0 | 0 | 6 | 0 |  |  |
| Road design to reduce car volume/speed | P | 28.6% | 0 | 2 | 0 | 0 | 0 | 0 | 0 |  |  |
| Traffic calming devices to reduce car volume/speed | P | 85.7% | 0 | 0 | 0 | 0 | 0 | 6 | 0 |  |  |
| Crossing aids for pedestrian / bicyclist | P | 71.4% | 0 | 0 | 0 | 0 | 0 | 6 | 0 |  |  |
| Street lighting for sidewalk, street shoulders, etc. | P | 97.6% | 0 | 0 | 0 | 0 | 0 | 0 | 7 |  |  |
| Sidewalk has heaves, cracks, broken sections, etc.^c^ | N | 25.0% | 0 | 2 | 0 | 0 | 0 | 0 | 0 | 1.50 | (N) |
| Sidewalk blocked by obstacles^c^ | N | 8.3% | 1 | 0 | 0 | 0 | 0 | 0 | 0 |  |  |
| **Facilities** |  |  |  |  |  |  |  |  |  |  |  |
| Public / recreational facilities | P | 11.9% | 0 | 2 | 0 | 0 | 0 | 0 | 0 | 1.33 | (P) |
| Public / recreational equipment | P | 9.5% | 1 | 0 | 0 | 0 | 0 | 0 | 0 |  |  |
| Playground equipment | P | 0.0% | 1 | 0 | 0 | 0 | 0 | 0 | 0 |  |  |
| **Aesthetics** |  |  |  |  |  |  |  |  |  |  |  |
| Attractive features (e.g., architecture, vegetation) | P | 64.3% | 0 | 0 | 0 | 0 | 5 | 0 | 0 | 5.50 | (P) |
| Comfort features (shade, trees, benches, etc.) | P | 78.6% | 0 | 0 | 0 | 0 | 0 | 6 | 0 |  |  |
| Air pollution | N | 0.0% | 1 | 0 | 0 | 0 | 0 | 0 | 0 | 4.17 | (N) |
| Noise pollution | N | 33.3% | 0 | 0 | 3 | 0 | 0 | 0 | 0 |  |  |
| Physical disorder (general) | N | 78.6% | 0 | 0 | 0 | 0 | 0 | 6 | 0 |  |  |
| Whole or broken beer or liquor bottles or can | N | 50.0% | 0 | 0 | 0 | 4 | 0 | 0 | 0 |  |  |
| Cigarette, cigar butts or discarded cigarette packages | N | 64.3% | 0 | 0 | 0 | 0 | 5 | 0 | 0 |  |  |
| Garbage, litter, or broken glass | N | 71.4% | 0 | 0 | 0 | 0 | 0 | 6 | 0 |  |  |
| **Signage** |  |  |  |  |  |  |  |  |  |  |  |
| Pedestrian or bicyclist friendly traffic signs | P | 61.9% | 0 | 0 | 0 | 0 | 5 | 0 | 0 | 2.00 | (P) |
| Share the road sign | P | 0.0% | 1 | 0 | 0 | 0 | 0 | 0 | 0 |  |  |
| Religious message | P | 14.3% | 0 | 2 | 0 | 0 | 0 | 0 | 0 |  |  |
| Political message | P | 2.4% | 1 | 0 | 0 | 0 | 0 | 0 | 0 |  |  |
| Fast food billboard | P | 7.1% | 1 | 0 | 0 | 0 | 0 | 0 | 0 |  |  |
| No trespassing / beware of dogs | N | 28.6% | 0 | 2 | 0 | 0 | 0 | 0 | 0 | 2.00 | (N) |
| Security warning | N | 21.4% | 0 | 2 | 0 | 0 | 0 | 0 | 0 |  |  |
| **Social Environment** |  |  |  |  |  |  |  |  |  |  |  |
| Children present in the street | P | 4.8% | 1 | 0 | 0 | 0 | 0 | 0 | 0 | 2.33 | (P) |
| Children engaged in active behavior | P | 4.8% | 1 | 0 | 0 | 0 | 0 | 0 | 0 |  |  |
| Teenagers/adults present in the street | P | 66.7% | 0 | 0 | 0 | 0 | 5 | 0 | 0 |  |  |
| Teenagers/adults engaged in active behavior | P | 66.7% | 0 | 0 | 0 | 0 | 5 | 0 | 0 |  |  |
| Older adults (>65 years) present in the street | P | 9.5% | 1 | 0 | 0 | 0 | 0 | 0 | 0 |  |  |
| Older adults (>65 years) engaged in active behavior | P | 9.5% | 1 | 0 | 0 | 0 | 0 | 0 | 0 |  |  |
| Stray dogs or animals (not squirrels or rabbits) | N | 11.9% | 0 | 2 | 0 | 0 | 0 | 0 | 0 | 2.00 | (N) |

Abbreviations: TRAIN, Travel Related Activity in Neighborhoods.

a. Observed features were arranged into two classes: positive (P) and negative (N). This classification was based on the expected direction of the relationship between each feature and physical activity behavior, as evidenced by findings in available literature.

b. C.I. scores were calculated separately for each class (positive vs. negative) in each domain. To calculate a C.I. score, we first dichotomized the observed categories for selected features, such that features with multiple categories were collapsed into “any observation” versus “no observation. Thereafter, we calculated the percentage of all the audited segments in the entire study area with “any observation” of these selected features. We then converted the calculated percentage to seven descriptive statements that ranged from “hardly ever” to “always”. For example, a feature that was observed in 0-10.9 percent of the audited segments was labeled "hardly ever," and so on. The seven descriptive statements were thereafter assigned scores, ranging from 1.00 for "hardly ever" (0%-10.9%) to 7.00 for "always" (91%-100%). We then calculated the average score for that class and maintained the min=1.00, max=7.00 average score (i.e., the C.I. score). Where positive relationship with physical activity is expected, a high C.I. score is indicative of prevalence of physical activity friendly features, whereas, for negative relationships, a high score indicates prevalence non-physical activity friendly features.

c. Data shown (percentages) are based on a subset of the audited 590 segments that are applicable to these particular audit questions. For example. Sidewalk continuity only applies to segments with sidewalks.

**Table B1.** Calculating the Composite Index (C.I.) scores for the **OST / South Union** super neighborhood (60 segments). Houston TRAIN Study, 2014.

| **Descriptive statements** | | | Hardly ever | Rarely | Occasionally | Half the time | Frequently | Usually | Always | Composite Index^b^ | |
| --- | --- | --- | --- | --- | --- | --- | --- | --- | --- | --- | --- |
| **Percent ranges** | | | 0-10.9 | 11-30.9 | 31-45.9 | 46-55.9 | 56-70.9 | 71-90.9 | 91-100 |  |  |
| **Scores** | | | 1.00 | 2.00 | 3.00 | 4.00 | 5.00 | 6.00 | 7.00 |  |  |
|  | P/N^a^ | (%) |  |  |  |  |  |  |  |  | |
| **Land Use** |  |  |  |  |  |  |  |  |  |  |  |
| Land use integration | P | 18.3% | 0 | 2 | 0 | 0 | 0 | 0 | 0 | 1.50 | (P) |
| Office building | P | 6.7% | 1 | 0 | 0 | 0 | 0 | 0 | 0 |  |  |
| Other services – e.g. beautician and lawyer | P | 15.0% | 0 | 2 | 0 | 0 | 0 | 0 | 0 |  |  |
| Fast food restaurants | P | 6.7% | 1 | 0 | 0 | 0 | 0 | 0 | 0 |  |  |
| Strip mall | P | 13.3% | 0 | 2 | 0 | 0 | 0 | 0 | 0 |  |  |
| Transportation facility | P | 8.3% | 1 | 0 | 0 | 0 | 0 | 0 | 0 |  |  |
| Place of worship | P | 8.3% | 1 | 0 | 0 | 0 | 0 | 0 | 0 |  |  |
| Park | P | 11.7% | 0 | 2 | 0 | 0 | 0 | 0 | 0 |  |  |
| Warehouses, factories, or industrial buildings | N | 8.3% | 1 | 0 | 0 | 0 | 0 | 0 | 0 | 3.00 | (N) |
| Auto shop | N | 5.0% | 1 | 0 | 0 | 0 | 0 | 0 | 0 |  |  |
| Parking lot or parking garage | N | 40.0% | 0 | 0 | 3 | 0 | 0 | 0 | 0 |  |  |
| Driveway (either residential or non-residential) | N | 95.0% | 0 | 0 | 0 | 0 | 0 | 0 | 7 |  |  |
| Abandoned building or vacant lot | N | 60.0% | 0 | 0 | 0 | 0 | 5 | 0 | 0 |  |  |
| Major transportation development (e.g. bridge, tunnel) | N | 5.0% | 1 | 0 | 0 | 0 | 0 | 0 | 0 |  |  |
| **Transportation** |  |  |  |  |  |  |  |  |  |  |  |
| Any alternative transportation modes | P | 60.0% | 0 | 0 | 0 | 0 | 5 | 0 | 0 | 4.06 | (P) |
| Any sidewalk facility | P | 60.0% | 0 | 0 | 0 | 0 | 5 | 0 | 0 |  |  |
| Sidewalk continuity; at least 1 side of street (both ends)^c^ | P | 69.4% | 0 | 0 | 0 | 0 | 5 | 0 | 0 |  |  |
| Curvilinear curbs; at least 1 side of street (both ends)**^c^** | P | 77.8% | 0 | 0 | 0 | 0 | 0 | 6 | 0 |  |  |
| Sidewalk coverage on left side of segment (>75%)^c^ | P | 88.9% | 0 | 0 | 0 | 0 | 0 | 6 | 0 |  |  |
| Sidewalk coverage on right side of segment (>75%)^c^ | P | 77.8% | 0 | 0 | 0 | 0 | 0 | 6 | 0 |  |  |
| Bike lane or marked shoulder (for bikes) | P | 3.3% | 1 | 0 | 0 | 0 | 0 | 0 | 0 |  |  |
| Bike racks present | P | 1.7% | 1 | 0 | 0 | 0 | 0 | 0 | 0 |  |  |
| Bus/transit stop present | P | 18.3% | 0 | 2 | 0 | 0 | 0 | 0 | 0 |  |  |
| Bus stop; covered shelter with/out bench^c^ | P | 54.5% | 0 | 0 | 0 | 4 | 0 | 0 | 0 |  |  |
| Non-concrete multi-use trails or paths | P | 8.3% | 1 | 0 | 0 | 0 | 0 | 0 | 0 |  |  |
| Posted speed limit | P | 11.7% | 0 | 2 | 0 | 0 | 0 | 0 | 0 |  |  |
| On-street parking | P | 50.0% | 0 | 0 | 0 | 4 | 0 | 0 | 0 |  |  |
| >3 directions at intersections | P | 86.7% | 0 | 0 | 0 | 0 | 0 | 6 | 0 |  |  |
| Road design to reduce car volume/speed | P | 30.0% | 0 | 2 | 0 | 0 | 0 | 0 | 0 |  |  |
| Traffic calming devices to reduce car volume/speed | P | 71.7% | 0 | 0 | 0 | 0 | 0 | 6 | 0 |  |  |
| Crossing aids for pedestrian / bicyclist | P | 46.7% | 0 | 0 | 0 | 4 | 0 | 0 | 0 |  |  |
| Street lighting for sidewalk, street shoulders, etc. | P | 93.3% | 0 | 0 | 0 | 0 | 0 | 0 | 7 |  |  |
| Sidewalk has heaves, cracks, broken sections, etc.^c^ | N | 30.6% | 0 | 2 | 0 | 0 | 0 | 0 | 0 | 2.00 | (N) |
| Sidewalk blocked by obstacles^c^ | N | 27.8% | 0 | 2 | 0 | 0 | 0 | 0 | 0 |  |  |
| **Facilities** |  |  |  |  |  |  |  |  |  |  |  |
| Public / recreational facilities | P | 16.7% | 0 | 2 | 0 | 0 | 0 | 0 | 0 | 1.67 | (P) |
| Public / recreational equipment | P | 10.0% | 1 | 0 | 0 | 0 | 0 | 0 | 0 |  |  |
| Playground equipment | P | 11.7% | 0 | 2 | 0 | 0 | 0 | 0 | 0 |  |  |
| **Aesthetics** |  |  |  |  |  |  |  |  |  |  |  |
| Attractive features (e.g., architecture, vegetation) | P | 70.0% | 0 | 0 | 0 | 0 | 5 | 0 | 0 | 5.50 | (P) |
| Comfort features (shade, trees, benches, etc.) | P | 90.0% | 0 | 0 | 0 | 0 | 0 | 6 | 0 |  |  |
| Air pollution | N | 1.7% | 1 | 0 | 0 | 0 | 0 | 0 | 0 | 3.67 | (N) |
| Noise pollution | N | 3.3% | 1 | 0 | 0 | 0 | 0 | 0 | 0 |  |  |
| Physical disorder (general) | N | 70.0% | 0 | 0 | 0 | 0 | 5 | 0 | 0 |  |  |
| Whole or broken beer or liquor bottles or can | N | 56.7% | 0 | 0 | 0 | 0 | 5 | 0 | 0 |  |  |
| Cigarette, cigar butts or discarded cigarette packages | N | 70.0% | 0 | 0 | 0 | 0 | 5 | 0 | 0 |  |  |
| Garbage, litter, or broken glass | N | 58.3% | 0 | 0 | 0 | 0 | 5 | 0 | 0 |  |  |
| **Signage** |  |  |  |  |  |  |  |  |  |  |  |
| Pedestrian or bicyclist friendly traffic signs | P | 43.3% | 0 | 0 | 3 | 0 | 0 | 0 | 0 | 1.40 | (P) |
| Share the road sign | P | 0.0% | 1 | 0 | 0 | 0 | 0 | 0 | 0 |  |  |
| Religious message | P | 8.3% | 1 | 0 | 0 | 0 | 0 | 0 | 0 |  |  |
| Political message | P | 5.0% | 1 | 0 | 0 | 0 | 0 | 0 | 0 |  |  |
| Fast food billboard | P | 6.7% | 1 | 0 | 0 | 0 | 0 | 0 | 0 |  |  |
| No trespassing / beware of dogs | N | 36.7% | 0 | 0 | 3 | 0 | 0 | 0 | 0 | 2.50 | (N) |
| Security warning | N | 20.0% | 0 | 2 | 0 | 0 | 0 | 0 | 0 |  |  |
| **Social Environment** |  |  |  |  |  |  |  |  |  |  |  |
| Children present in the street | P | 15.0% | 0 | 2 | 0 | 0 | 0 | 0 | 0 | 3.00 | (P) |
| Children engaged in active behavior | P | 11.7% | 0 | 2 | 0 | 0 | 0 | 0 | 0 |  |  |
| Teenagers/adults present in the street | P | 70.0% | 0 | 0 | 0 | 0 | 5 | 0 | 0 |  |  |
| Teenagers/adults engaged in active behavior | P | 68.3% | 0 | 0 | 0 | 0 | 5 | 0 | 0 |  |  |
| Older adults (>65 years) present in the street | P | 18.3% | 0 | 2 | 0 | 0 | 0 | 0 | 0 |  |  |
| Older adults (>65 years) engaged in active behavior | P | 13.3% | 0 | 2 | 0 | 0 | 0 | 0 | 0 |  |  |
| Stray dogs or animals (not squirrels or rabbits) | N | 8.3% | 1 | 0 | 0 | 0 | 0 | 0 | 0 | 1.00 | (N) |

Abbreviations: TRAIN, Travel Related Activity in Neighborhoods.

a. Observed features were arranged into two classes: positive (P) and negative (N). This classification was based on the expected direction of the relationship between each feature and physical activity behavior, as evidenced by findings in available literature.

b. C.I. scores were calculated separately for each class (positive vs. negative) in each domain. To calculate a C.I. score, we first dichotomized the observed categories for selected features, such that features with multiple categories were collapsed into “any observation” versus “no observation. Thereafter, we calculated the percentage of all the audited segments in the entire study area with “any observation” of these selected features. We then converted the calculated percentage to seven descriptive statements that ranged from “hardly ever” to “always”. For example, a feature that was observed in 0-10.9 percent of the audited segments was labeled "hardly ever," and so on. The seven descriptive statements were thereafter assigned scores, ranging from 1.00 for "hardly ever" (0%-10.9%) to 7.00 for "always" (91%-100%). We then calculated the average score for that class and maintained the min=1.00, max=7.00 average score (i.e., the C.I. score). Where positive relationship with physical activity is expected, a high C.I. score is indicative of prevalence of physical activity friendly features, whereas, for negative relationships, a high score indicates prevalence non-physical activity friendly features.

c. Data shown (percentages) are based on a subset of the audited 590 segments that are applicable to these particular audit questions. For example. Sidewalk continuity only applies to segments with sidewalks.
